# Effects of processing conditions on stability of immune analytes in human blood

**DOI:** 10.1038/s41598-020-74274-8

**Published:** 2020-10-15

**Authors:** Andres Gottfried-Blackmore, Samuel J. S. Rubin, Lawrence Bai, Sheun Aluko, Yujun Yang, Walter Park, Aida Habtezion

**Affiliations:** 1 Division of Gastroenterology and Hepatology, Department of Medicine, Stanford University School of Medicine, Alway Building M211, 300 Pasteur Drive, Stanford, CA 94305 USA; 2 Immunology Program, Stanford University School of Medicine, 1215 Welch Road, Modular B, Stanford, CA 94305 USA; 3 Biomedical Informatics, Stanford University School of Medicine, 291 Campus Drive, Li Ka Shing Building, Stanford, CA 94305 USA

**Keywords:** Biomarkers, Immunology, Applied immunology, Translational immunology

## Abstract

Minimizing variability in collection and processing of human blood samples for research remains a challenge. Delaying plasma or serum isolation after phlebotomy (processing delay) can cause perturbations of numerous analytes. Thus, a comprehensive understanding of how processing delay affects major endpoints used in human immunology research is necessary. Therefore, we studied how processing delay affects commonly measured cytokines and immune cell populations. We hypothesized that short-term time delays inherent to human research in serum and plasma processing impact commonly studied immunological analytes. Blood from healthy donors was subjected to processing delays commonly encountered in sample collection, and then assayed by 62-plex Luminex panel, 40-parameter mass cytometry panel, and 540,000 transcript expression microarray. Variance for immunological analytes was estimated using each individual’s baseline as a control. In general, short-term processing delay led to small changes in plasma and serum cytokines (range − 10.8 to 43.5%), markers and frequencies of peripheral blood mononuclear cell phenotypes (range 0.19 to 3.54 fold), and whole blood gene expression (stable for > 20 K genes)—with several exceptions described herein. Importantly, we built an open-access web application allowing investigators to estimate the degree of variance expected from processing delay for measurements of interest based on the data reported here.

## Introduction

The advent of high-dimensional acquisition technology has led to an increase in human translational and basic research. Venous blood is one of most common biospecimens collected in clinical research. Blood continues to be the most common biospecimen assayed in human immunology research, given its relative ease of collection and processing. Blood includes a variety of fractions, including plasma, serum, white blood cells, and red blood cells. Blood components are labile, and their integrity requires timely processing. The allowable time varies by the component of interest and its stability. Therefore, blood collection and processing is dictated by the intended analysis, as no single collection method will meet all needs rev by^1^. Prior studies have established that (a) live cells are stable at room temperature (RT) for up to 48 h^2^; (b) immediate separation of plasma from cells (< 24 h at RT or 4 °C) is required to limit protein/peptide degradation^3^; (c) a 24 h time delay at 4 °C between sample collection and processing is acceptable and does not adversely affect the quality of extracted DNA^4^; and (d) delayed processing > 12 h at 4 °C is correlated with RNA degradation for both plasma and buffy coat samples^5^.

Despite implementation of these findings into some standard operating procedures, the methods used to collect, process, and store blood samples can vary widely within and across different institutions and laboratories. Even within a laboratory, investigators tend to pool results from samples subjected to different processing delays (i.e., overnight at 4 °C vs. 2 h at RT), given the inherent delays from phlebotomy to centrifugation in clinical research. Variations in blood handling markedly affect detection of clinically relevant diagnostic biomarkers. In particular, delaying plasma or serum isolation after phlebotomy can cause perturbations of numerous small molecule analytes^6^ and affect metabolite and proteomic studies used in research and clinical practice^3,7,8^.

Recently, it was reported that time delays in blood processing lead to variability in measurements of human monocyte subsets and subset-specific monocyte-platelet aggregates^9^, as well as myeloid-derived suppressor cells^10^. The effect of processing delay on select cytokines was also reported for different anticoagulants and temperatures^11,12^. Further research is needed to characterize the effects of processing delays on serum, plasma, and cellular analytes. The goal of this study was to determine how common blood sample processing delays are a source of variation in immunological research endpoints. We hypothesized that RNA, plasma, serum, and peripheral blood mononuclear cell (PBMC) processing delays impact commonly studied immunological endpoints such as transcript and protein levels, as well as PBMC viability and phenotype. Blood from healthy donors was systematically subjected to processing delays and temperature conditions that are common in blood processing for research, and then assayed by 62-plex Luminex, 40-parameter mass cytometry, and gene expression microarray. The results from this study will help guide investigators using human blood samples to minimize pre-analytical variability and identify threshold delay times for particular endpoints of interest.

## Results

**Figure 1:**
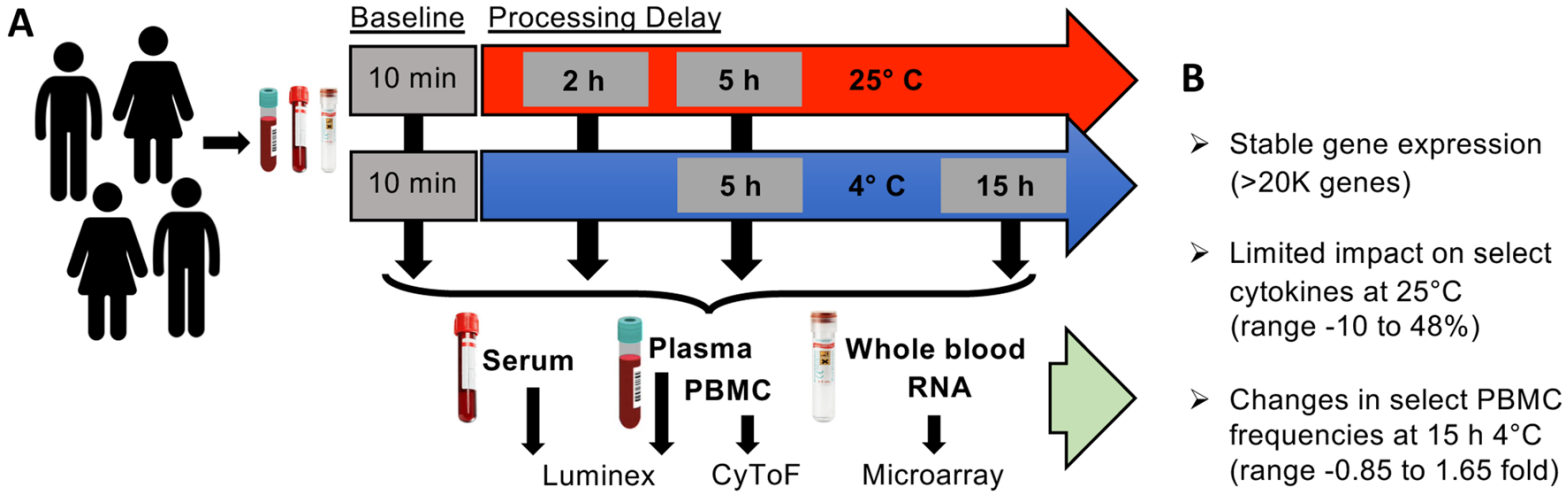
Effects of processing conditions on stability of immune analytes in human blood. (**A**) Experimental design testing the effects of short term processing delays on immunological analytes. (**B**) Summary results for each of the three assays performed.

**Figure 2:**
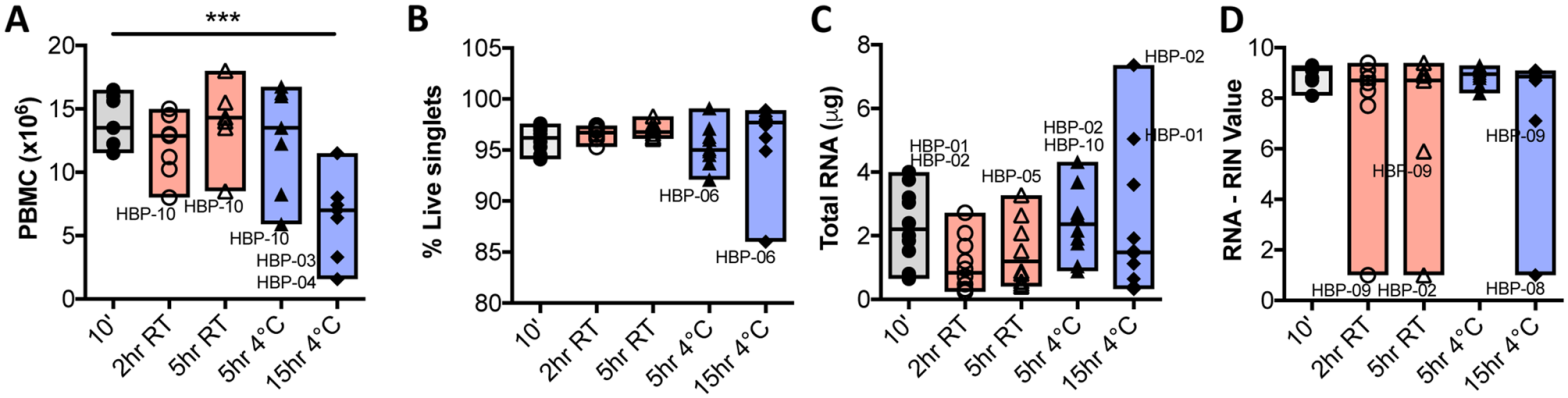
Human blood processing short time-delay has minimal effects on cell viability and RNA yields. (**A**) Peripheral blood mononuclear cell (PBMC) viable (trypan blue negative) counts after ficoll separation show decreased counts at 15 h 4 °C. (**B**) Frequency of live singlets in cryopreserved, thawed, stimulated, and stained PBMCs analyzed by CyTOF for unstimulated samples grouped by processing condition. (**C**) Total RNA yields at all conditions assayed. (**D**) RNA RIN values at all conditions tested. In all plots, individual outliers were labeled. (n = 10 per condition; *p ≤ 0.05; repeated-measures One-Way ANOVA and BY test for multiple comparisons).

Ten subjects were enrolled into the study, equally matched by sex and age (women mean age 35.6^+/−16.1^ years, men 33.8^+/−9.5^ years), and the majority were non-Hispanic white (Table 1). From each subject, 5 blood samples were obtained to test all processing conditions including a baseline condition as an intraindividual control, yielding 50 samples for immune analyte analyses (Fig. 1). Live PBMC cell counts decreased at 15 h 4 °C (Fig. 2A). The frequency of live cells in samples analyzed by CyTOF was high and stable (Fig. 2B; median 97–95% for unstimulated and stimulated samples respectively; range 99–85%). RNA yields and quality at most time points was stable (Fig. 2C,D; Supplemental Fig. 1).

**Table 1:** Subject characteristics.

ID	Age	Sex	Race
HBP 01	63	F	Asian
HBP 02	41	M	White
HBP 03	24	M	White
HBP 04	25	F	White
HBP 05	35	M	African
HBP 06	37	F	White
HBP 07	29	F	Asian
HBP 08	45	M	White
HBP 09	24	M	Asian
HBP 10	24	F	White

### Whole blood transcriptomes are largely stable over time

To analyze transcriptomic changes in whole blood over time, we selected the Paxgene format because it ensures RNA stability once blood is collected into its vacutainers. Care was taken to follow the manufacturer’s instructions in filling the Paxgene tubes from our previously collected blood samples in sterile heparin sulfate vacutainers as if they had been filled from a freshly drawn blood sample. This allowed estimation of transcriptomic contributions to changes observed over processing conditions.

**Figure 3:**
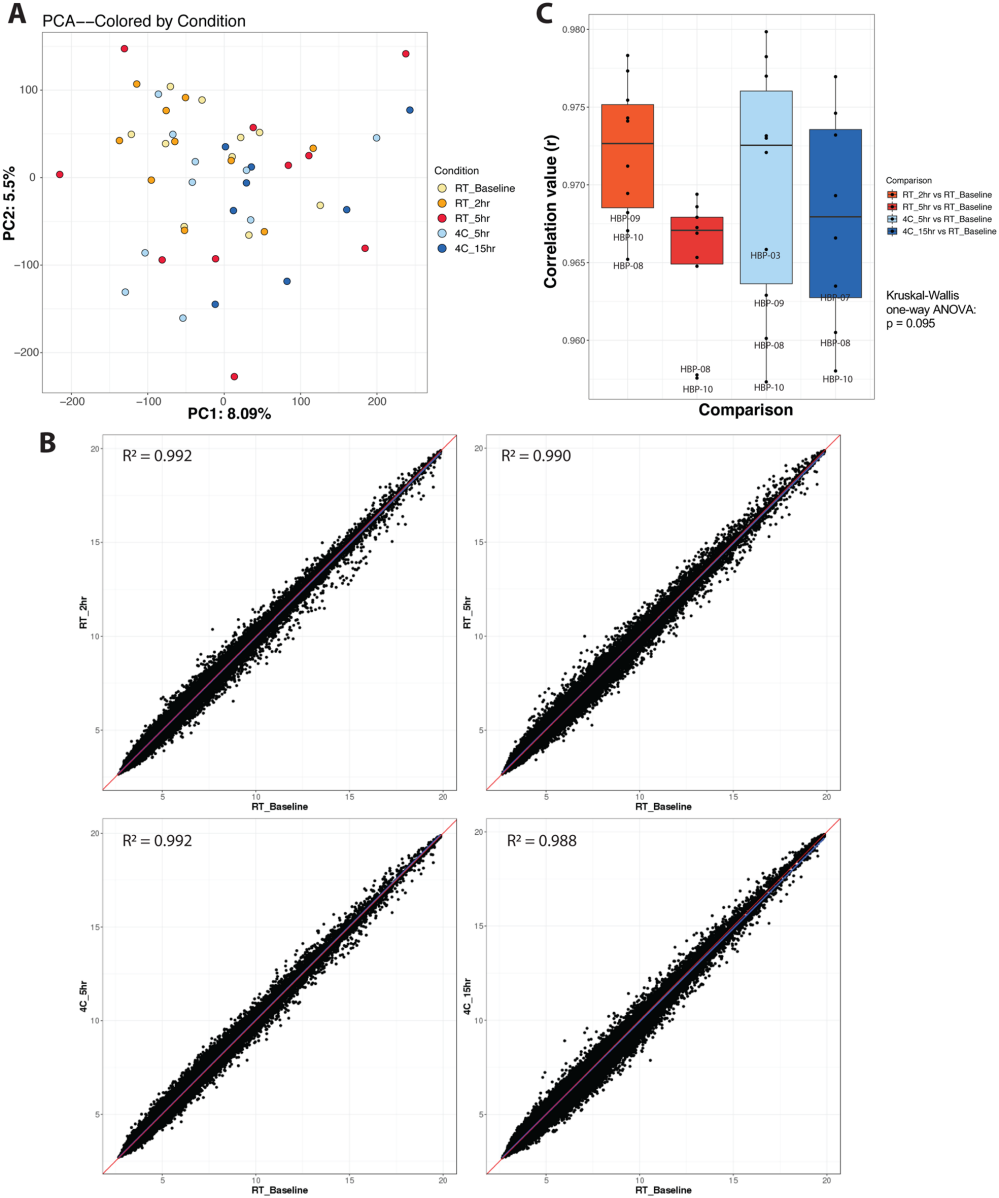
Whole blood transcriptomes are largely stable over time. (**A**) PC analysis of samples containing all conditions tested. Axis percentages indicate variance contribution. (**B**) Correlation scatterplots between baseline and each condition tested. Values were averaged for each probe for a particular condition. (**C**) Paired correlation analysis between each condition compared to baseline for all individual subjects (p = 0.095; Kruskal–Wallis One-Way ANOVA). (n = 10 per condition, except n = 8 for the 15 h time point as two samples, HBP-04 and 09, did not meet ThermoFisher microarray QC metrics).

We used an unbiased approach to evaluate any transcriptomic differences over time and temperature. Principal components analysis (PCA) did not reveal any pattern between time delay and variance of gene expression (Fig. 3A); additionally, there was no pattern associated with other independent variables, including age, sex, race, or individual subject (Supplemental Fig. 2). Pearson correlations between mean baseline expression and each subsequent mean condition were highly concordant (Fig. 3B). Upon closer inspection, paired correlation analysis for each individual subject and their respective timepoint delay revealed somewhat lower correlation values at the 5 h RT timepoint, but the trend was not statistically significant (*p* = 0.094, Kruskal–Wallis one-way ANOVA; Fig. 3C).

**Figure 4:**
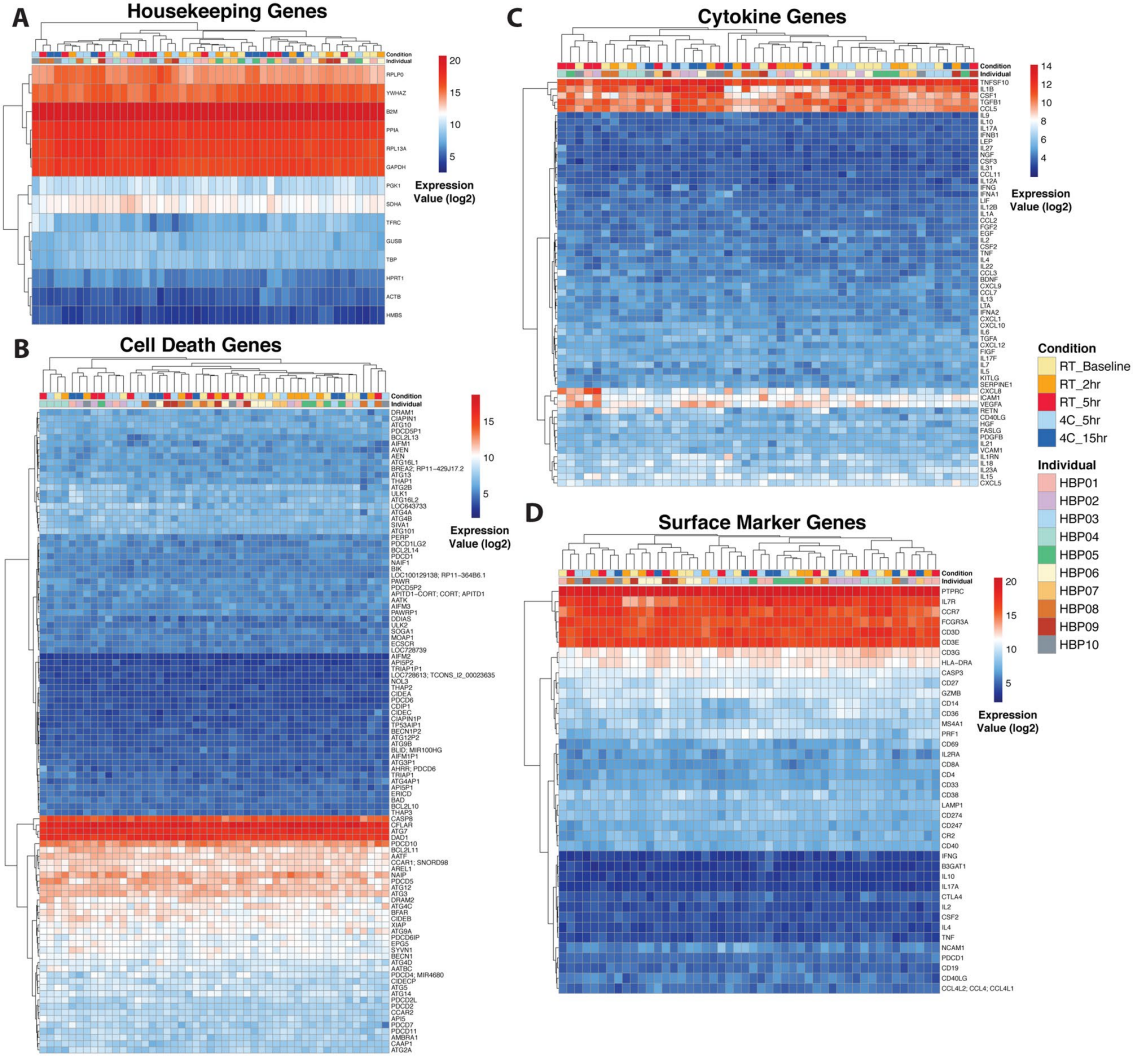
Whole blood housekeeping, cell death, and immune gene expression profiles are unaffected by different time and temperature conditions. Gene expression heatmaps were generated across (**A**) common housekeeping genes; (**B**) apoptosis/cell death genes, as described in their gene function description (see Methods); (**C**) genes corresponding to proteins measured by Luminex ; and (**D**) genes corresponding to proteins measured by CyTOF. Hierarchical clustering analysis was performed for each heatmap. (n = 10 per condition, except n = 8 for the 15 h time point as two samples, HBP-04 and 09, did not meet ThermoFisher microarray QC metrics; see Supplemental Fig. 1C).

Due to the noticeable decrease in total viable cell count over time (Fig. 2A), we evaluated the stability of housekeeping genes, cell death genes, cytokines, and cell surface markers. Most housekeeping genes had minimal variability across time points (Fig. 4A). Cell death and apoptosis genes were largely stable (Fig. 4B). Several transcripts encoding cytokines were more variable than others (Fig. 4C), but unsupervised hierarchical clustering did not reveal a specific time or temperature pattern that explained this variance. Transcripts encoding markers measured by CyTOF were also generally unchanged across processing conditions (Fig. 4D). Furthermore, Kruskal–Wallis tests across the five delay conditions revealed no statistically significant genes in any of the gene sets (Supplemental Table 1).

### Serum and plasma cytokines are differentially impacted by temperature and processing delays

**Figure 5:**
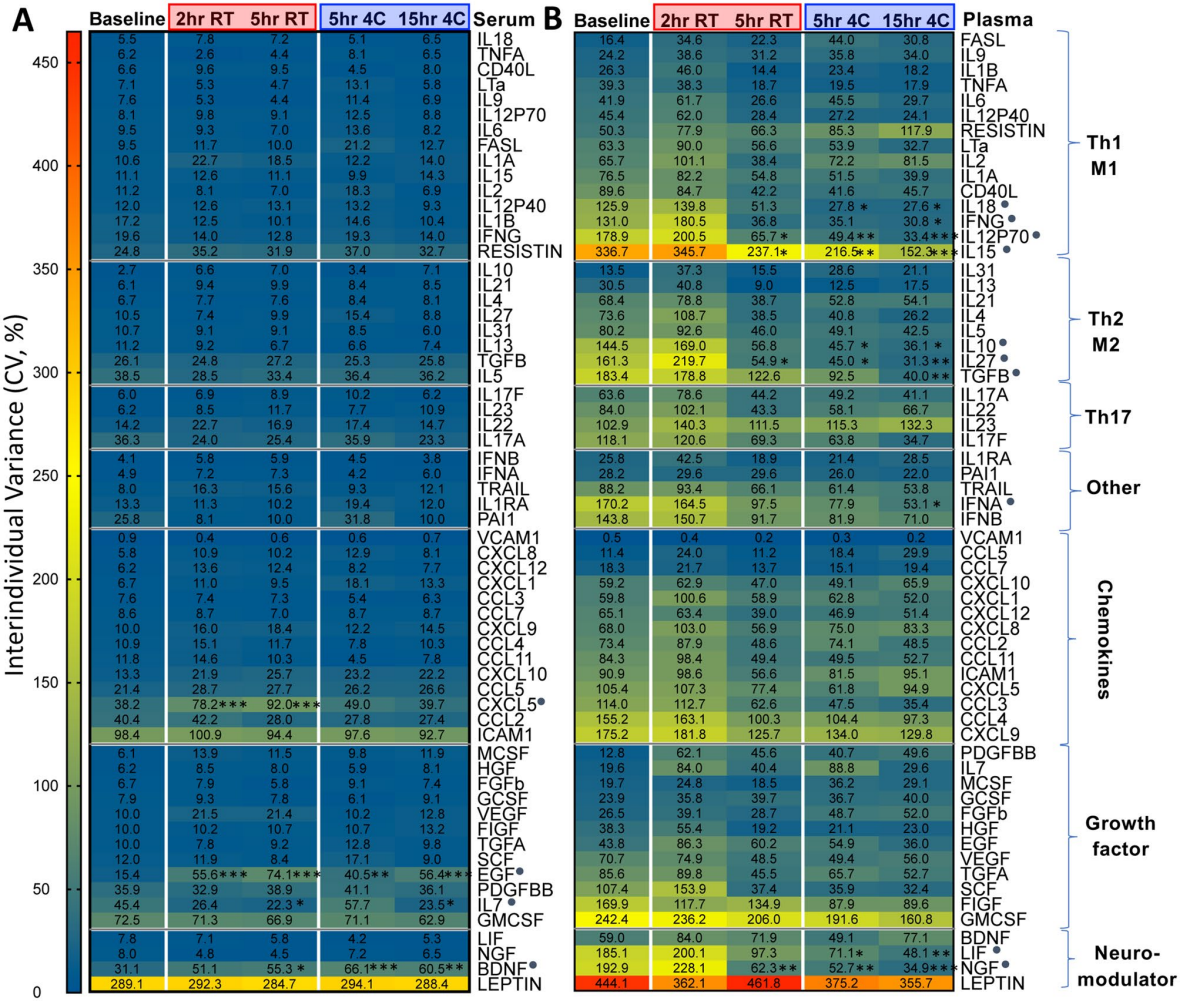
Serum cytokines have reduced variance, and time delay reduces variance in plasma cytokines. Heatmaps showing interindividual variance for all 62 cytokines assayed, grouped by putative functional groups, in serum (**A**) and plasma (**B**). Cytokines were ranked by variance at baseline, and the ones with processing delay-associated significant differences were marked with a grey dot. (n = 10 per condition; *p < 0.05, **p < 0.01, ***p < 0.001, ****p < 0.0001; Two-Way ANOVA with Bonferroni posthoc test for multiple comparisons).

Consistent with prior reports^3^, plasma and serum cytokines were poorly correlated with each other, regardless of processing delay (Supplemental Fig. 3). Serum cytokines had negligible interindividual variance, in contrast to plasma (Fig. 5). Processing delay at 4 °C in plasma was associated with decreased variance (Fig. 5B).

**Figure 6:**
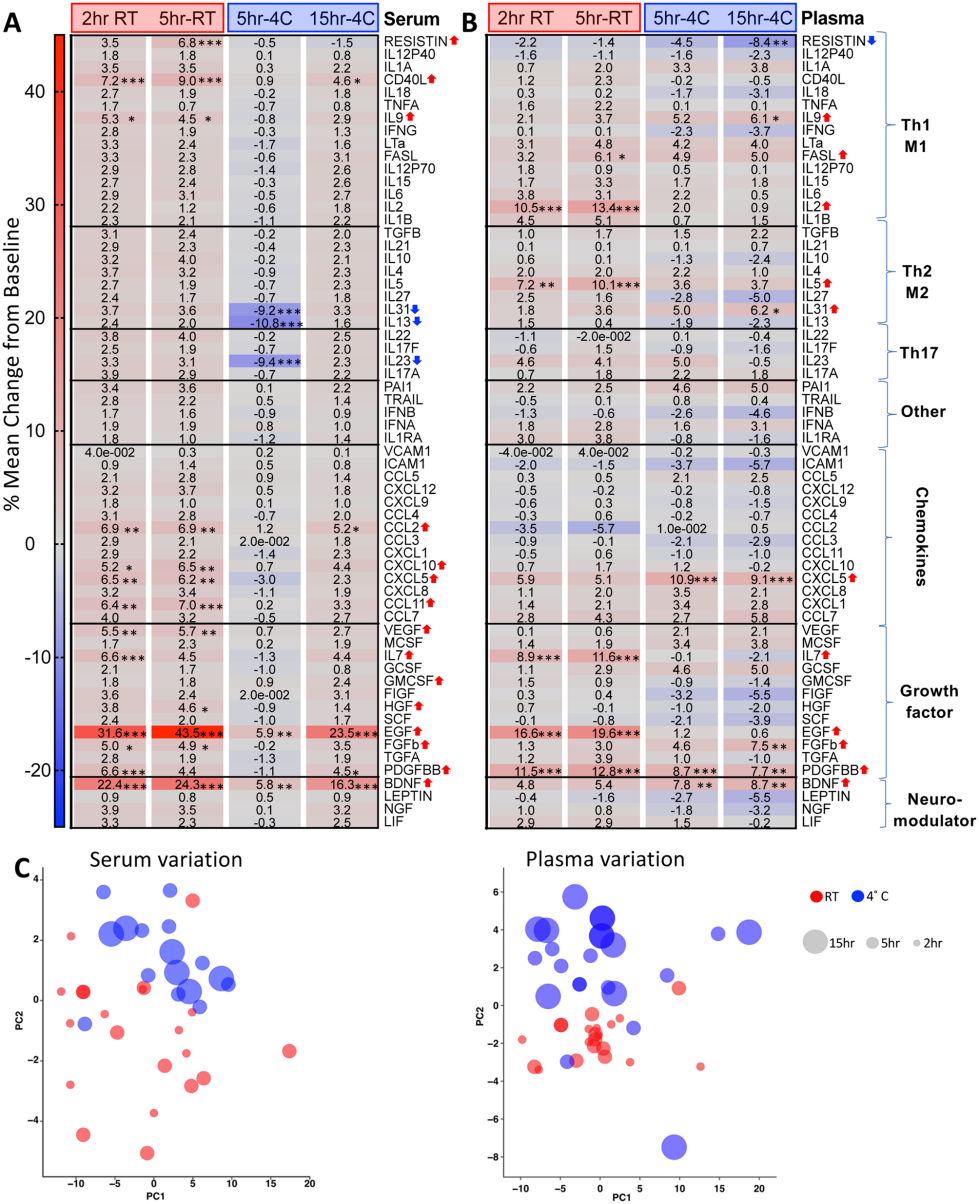
Serum and plasma cytokines are differentially impacted by temperature and processing delays. Heatmaps showing % change in 62 cytokines as a function of each individual’s baseline in serum (**A**) and plasma (**B**). Mean % change was calculated by dividing each time point by the baseline time point for each respective individual and cytokine, multiplying by 100, subtracting 100 to get the % change, and then computing the mean of the cohort’s % change. Cytokines grouped by putative functional groups and ranked by abundance in blood for each subgroup. (**C**) PCA plots showing effect of temperature (color code) and time (circle size) for each individual on aggregate cytokine variation in serum and plasma. (n = 10 per condition; *p < 0.05, **p < 0.01, ***p < 0.001; repeated-measures Two-Way ANOVA Bonferroni posthoc test for multiple comparisons).

When controlling for interindividual variance, serum was more susceptible to processing delay, with 15 of 62 (24%) cytokines being significantly increased and 3 of 62 (4.8%) cytokines being decreased over time (Fig. 6A). Plasma cytokines showed 11 of 62 (18%) and 1 of 62 (1.6%) cytokines increased, and decreased respectively, over time (Fig. 6B). The magnitude of change was relatively small overall, with most circulating cytokines showing little to no change (serum median change − 0.4 to 3.1%, range − 10.8 to 43.5%; plasma median change 0.1 to 1.7%, range − 8.4 to 19.6%) (Fig. 6). Many cytokines were increased at RT processing delay for both serum and plasma. In contrast, refrigeration for the same 5 h time delay at 4 °C reduced or prevented serum cytokine increases (Resistin, CD40L, IL9, chemokines, EGF, BDNF), and in some instances was associated with decreased cytokine levels (IL31, IL13, and IL23) (Fig. 6A,C). In plasma, refrigeration was associated with reduced cytokine increases (IL2, IL5, IL7), and in some instances with greater cytokine levels (IL9, IL31, CXCL5, BDNF) (Fig. 6B,C). Thus, plasma and serum cytokine profiles were distinct.

Given that published studies involving blood sampling almost never include an intraindividual control for processing delay, we also calculated the effect of processing delays as a function of the group median baseline values. This analysis showed much larger magnitude changes (serum range − 77 to 414%; plasma range − 38 to 159%) (Supplemental Fig. 4). The overall increase in cytokines observed in plasma was maintained in this analysis. Serum showed an overall decreased trend with processing delay due to two individuals who increased the baseline median disproportionately.

In sum, most serum and plasma cytokines measured in the 62-plex panel were stable for processing delays up to 15 h. However, RT processing delay had an overall greater impact compared to refrigerated processing delay. There was greater interindividual variation in plasma samples, and select cytokines that were downregulated by processing delay had a sex bias towards females (not shown). These results suggest that processing delays up to 15 h under refrigerated conditions are acceptable for the measurement of most circulating cytokines with several noted exceptions, and that serum preparations yield much smaller group variance than plasma preparations.

### Select cellular features are impacted by processing delay and refrigeration

We manually gated 284 cellular frequencies (Supplemental Fig. 5), of which 19 were significantly different between baseline and 5 h RT or 4 °C or baseline and 15 h 4 °C delayed processing conditions after adjusting for multiple comparisons (Supplemental Table 2). When comparing 64 cytokine-positive cell frequencies derived from IL2, TNFα, and IFNγ in canonical cytokine-producing cell populations (based on trends observed in Luminex data), 21 significant differences were identified for stimulated cells only, amongst which CD4^+^ and CD8^+^ T cells were highly represented (Supplemental Table 3). Significantly different T, NKT, and NK cell populations, but not monocytes, identified here had increased cytokine production ability with greater time delay and refrigeration, which could represent a direct effect of processing delay and temperature or an indirect effect mediated by selective survival of cells capable of cytokine production in response to stimulation.

**Figure 7:**
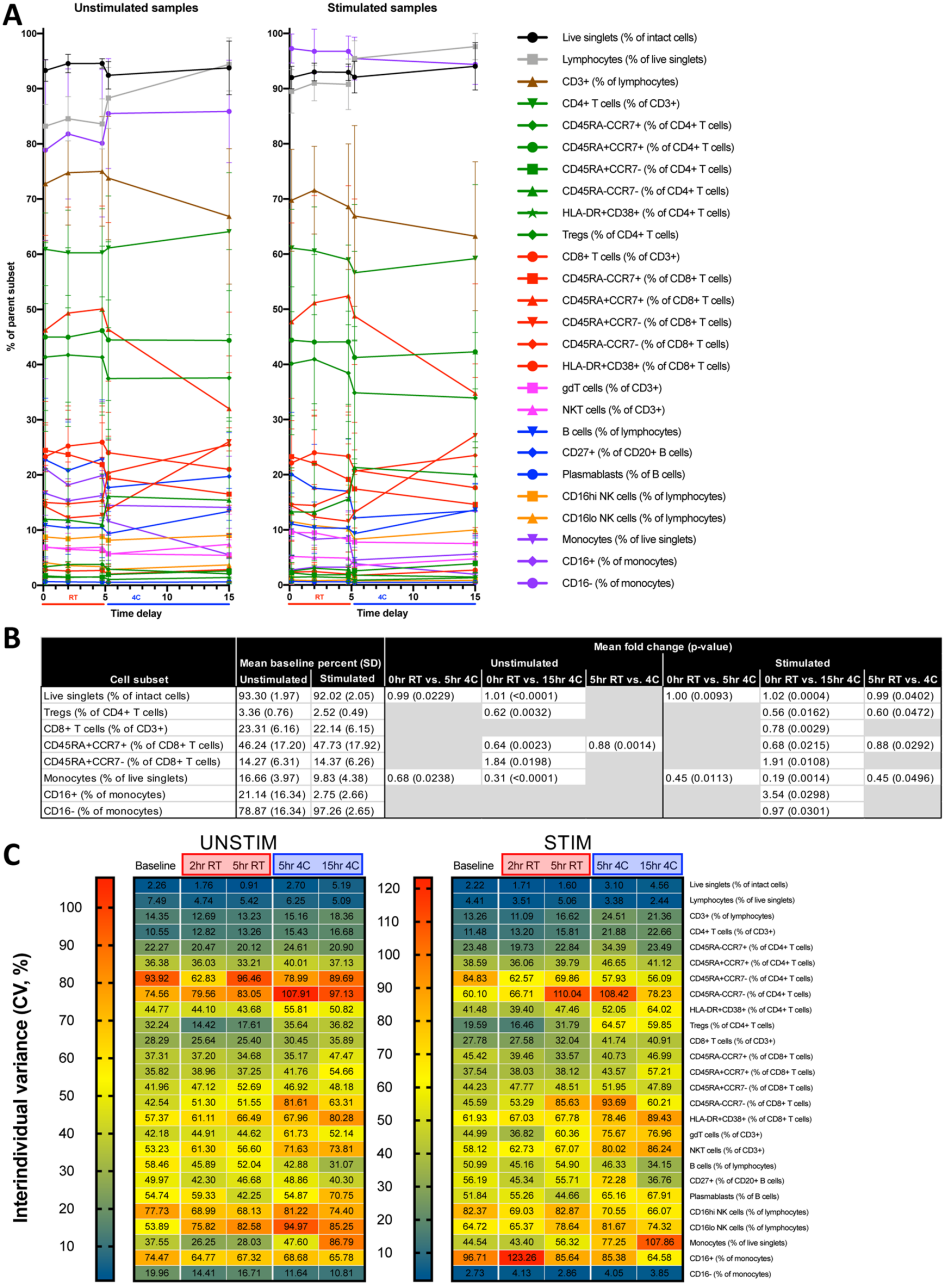
Select cellular features are impacted by processing delay and refrigeration. (**A**) 26 canonical cell populations were gated and traced between five conditions for unstimulated (left) and stimulated (right) samples. Each condition was compared to baseline for unstimulated or stimulated samples, and (**B**) significant differences were identified. RT was also compared to 4C for the 5 h conditions. (**C**) Interindividual variance (CV) for 26 canonical cell populations was calculated for each five conditions for unstimulated (left) and stimulated (right) samples. Statistics: paired two-tailed Student’s T-test with BH method correction for multiple comparisons. Mean fold change was calculated by taking the average of the fold change for each individual. (n = 10 per condition).

Due to the stringency of adjustment for multiple comparisons in the analysis of hundreds of gated cell parameters, we identified 26 canonical cell populations frequently reported in immunological studies. When comparing each condition to baseline, and the two 5 h conditions to each other, for these 26 canonical cell populations, the frequencies of monocytes, lymphocytes, and several widely studied T cell subsets were significantly different between baseline and 5 h and 15 h conditions, as well as between the two 5 h conditions (Fig. 7A,B). Cell stimulation prior to CyTOF analysis appeared to synchronize cells within conditions, yielding lower interindividual variance for many of the 26 canonical populations (Fig. 7C), and more significant differences when comparing stimulated samples between conditions. We calculated 356 median signal intensities across these 26 canonical cell populations, of which only 11 were significantly different between processing conditions (Supplemental Table 4). These findings suggest that highly relevant immunological changes to PBMCs manifest by 5 h.

To investigate the role of selective cell death in determining differential cell frequencies across conditions, we gated cleaved caspase 3 positive subfractions of the 26 canonical cell populations (caspase 3^+^; increased cleavage indicates apoptosis). We found 15 significantly affected subpopulations (Supplemental Table 5) with differential caspase 3 cleavage, which overlapped with the 26 canonical populations (Fig. 7A,B), suggesting that selective apoptosis may drive observed differences in cell frequency.

To explore other cellular parameters in a manner unbiased by our manual gating, we conducted viSNE and CITRUS analysis based on live singlets (Supplemental Figs. 6 and 7). This approach confirmed that most differences occurred when comparing 15 h conditions to baseline. viSNE analysis revealed volatility in the monocyte compartment, while CITRUS analysis highlighted changes to specific T cell populations and expression of checkpoint receptors and ligands. In contrast to our manual gating approach which identified parameters that increased over time, this unbiased approach predominantly identified parameters that decreased over time and also included cell populations not otherwise gated.

To further validate our analysis, CyTOF data was reanalyzed with the independent Astrolabe software^13^. Results were largely consistent with our analysis despite different gating hierarchies and significance testing methods (Supplemental Fig. 8). Differences in frequencies of monocytes and naive CD8 + T cells across time and temperature conditions were comparable between both approaches. Astrolabe also identified subsets such as conventional DCs as being differentially represented between sample conditions. In the “profiling” level, Astrolabe software identified several non-canonical cell subsets differentially represented between conditions.

**Figure 8:**
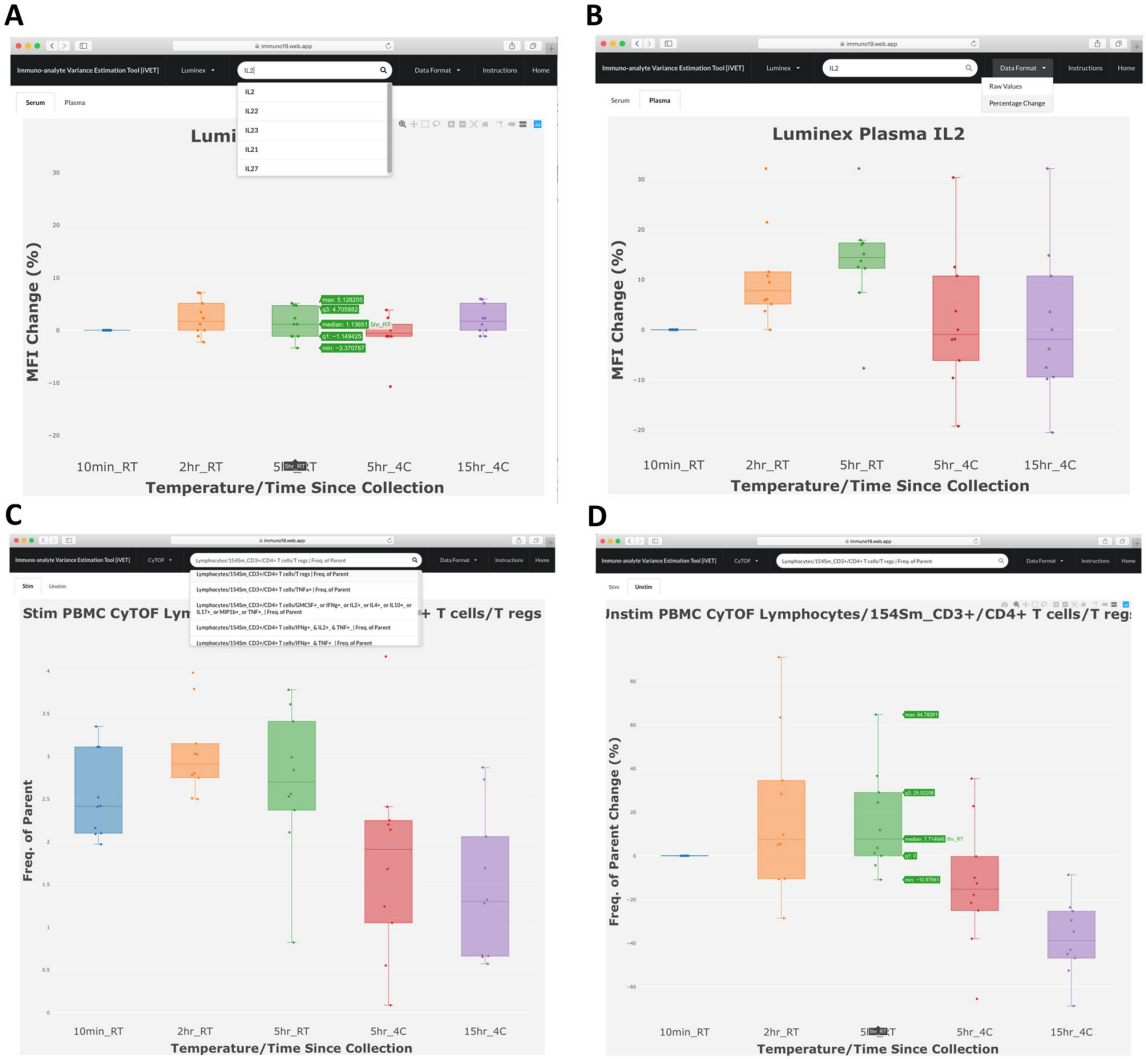
Immuno-analyte Variance Estimation Tool (iVET) web interface. Example plots showing IL-2 Luminex data as % change from baseline in serum (**A**) or plasma (**B**). In (**A**), cursor on 5 h RT condition highlights median, interquartile range, and max, min values. (**C**) CyTOF search showing all cell subsets with “CD4” in dropdown menu, with selection of CD4^+^ Treg cells under stimulated (**C**) and unstimulated (**D**) conditions.

### Immuno-analyte Variance Estimation Tool (iVET) https://immuno19.web.app/

iVET is a search engine using our Microarray, Luminex or CyTOF data. Selected analytes are displayed in an interactive bar plot graph showing individual data points with median, maximum and minimum values. Luminex data is sorted into “Serum” or “Plasma” tabs (Fig. 8A,B), whereas CyTOF data is sorted into “Stimulated” or “Unstimulated” tabs. Results can be displayed as normalized raw data or % change from baseline (Fig. 8C,D). The search bar displays dropdown lists including all analytes containing the search term of interest (Fig. 8A,C). Each plot displays a menu of options to pan, zoom, scale, and apply other visualization tools (Fig. 8A,D). Plots can be downloaded as PNG files. Figure 8 shows an example of how iVET can easily display the effect of processing delay and temperature on IL-2 in serum versus plasma (Fig. 8A,B), showing greater variance in plasma. Bottom panels show CD4^+^ Treg cells by CyTOF under stimulated and unstimulated conditions (Fig. 8C,D), demonstrating how refrigerated processing delay decreased Treg frequency. Thus, iVET can generate visual representations of immune analytes in our study and allow investigators to estimate expected variance in their transcript, cytokine, or cell of interest as a function of processing conditions.

## Discussion

Human blood processing delay is one of the preanalytical variables most difficult to control compared to other parameters, such as demographic or clinical variables, choice of anticoagulant, methodological parameters, sample storage temperature, etc. We compared four scenarios of processing delay commonly practiced in immunology laboratories, compared RT to refrigeration, and performed a multiparametric comparison of plasma and serum. Importantly, we incorporated intraindividual controls for each sample by including a baseline condition for each subject. Our analysis encompassed whole blood gene expression, cytokine levels, and single-cell cytometry. Although most immune markers were largely stable following ≤ 15 h delays among all three methods, we identified some analytes volatile to processing delay, which should be a key consideration in future studies. Using the data generated from this project, we developed Immuno-analyte Variance Estimation Tool (iVET), a web application that allows investigators to estimate the degree of variance expected from processing delay for measurements of interest. This application provides an accessible platform for rapid data visualization and interpretation.

Clariom-D microarray analysis allowed us to assess the impact of processing delay on gene expression including transcript variants. Gene expression profiles were largely stable across time points and temperature conditions. Previous small studies suggested expression differences with blood processing delay in signal transduction, transcription, cell cycle arrest, and chemotaxis genes^14,15^, but these were mostly unchanged in our data, except CD83, CXCL8 and CD69, which showed a non-significant increased trend at 5 h RT. These discrepancies could be due to extended processing delays > 24h^15^ and/or differences between whole blood RNA versus PBMCs following Ficoll separation^14^. Our RNA yields support refrigeration of blood after phlebotomy and are consistent with prior studies showing no difference in plasma RNA concentrations obtained from uncentrifuged anticoagulated blood left at 4 °C up to 24 h^16^. Overall, transcript abundance was stable over our short-term processing delay conditions, both at RT and at 4 °C. This suggests that short-term delays (within 15 h) in processing blood for gene expression studies may not affect downstream analyses, and that differences observed in blood cytokines and cells may be largely determined by post-transcriptional events. Furthermore, platforms such as Paxgene, which allow for immediate sample RNA stabilization at the time of phlebotomy, can be used in clinical studies to circumvent these potential confounders.

Cytokines measured by Luminex showed that serum was more susceptible to processing delay compared to plasma, with most effects occurring at RT conditions. However, the magnitude of intra-individual change in serum cytokines was relatively small (max 43%), and serum had small inter-individual variance, which should help lower sample sizes necessary for power requirements in blood cytokine studies. Compared to serum, plasma cytokines showed higher interindividual variance, which was modestly reduced by refrigeration. The greater impact of processing delay at RT was consistent with results from Hennø et al., who showed that in various plasma preparations several cytokines were significantly increased at 1–4 h RT compared to 4°C^12^.

Overall, the choice to use plasma versus serum should rest on whether cell analysis is desired. There is poor correlation between serum and plasma, and these should not be used interchangeably for cytokine or proteomic analyses. When cell analysis is not necessary, the increased stability and decreased variance of serum may make this the preferred sample, although perhaps less physiologic. When cell analysis is desired, collection of plasma is incident and requisite if only one type of phlebotomy tube may be filled. When both plasma and cells are required, minimizing time delay is an evident goal, but the decision to refrigerate or keep samples at RT is less straightforward. For many plasma analytes, refrigeration at 4 °C is preferable for stability. However, there are conflicting reports in the literature as to whether blood sample refrigeration is beneficial or detrimental for flow cytometry analysis of different PBMC subsets^17^. We found that frequencies of 3 of 26 canonical PBMC subsets surveyed by CyTOF following a 5 h processing delay at 4 °C were significantly decreased compared to 5 h RT, including live singlets, monocytes, and Treg. CD45RA^+^CCR7^+^ CD8^+^ T cells were also significantly decreased in 4 °C compared to RT. Moreover, Caspase 3^+^ as percent of lymphocytes was 62.5% higher at 4 °C compared to RT for stimulated samples, indicating an increased induction of apoptosis. Thus, it is not clear whether refrigeration of anticoagulated blood samples is advantageous for downstream cellular assays, but there are effects of temperature. The decision whether to keep samples at RT or 4 °C could be guided by specific analytes of interest using these datasets and the iVET resource.

To our knowledge this is the first comprehensive study to assess the impact of processing delays on canonical and non-canonical leukocytes found in PBMC fractions. We focused on canonical cell populations that were most abundant, and less so on rare populations that may be more subject to statistical noise. Our findings of decreased monocytes with refrigeration were consistent with Ji et al.^9^. Most significant changes appreciated by CyTOF were observed for processing delays of 15 h at 4 °C, involving high level parameters such as the frequency of live single cells or monocytes versus lymphocytes, as well as widely studied subpopulations such as Treg and CD8^+^ T cells. The differences between room temperature and refrigeration as compared at the 5 h timepoint were minimal, with similarly high cell viability, suggesting that the two processing delay conditions are favorable for downstream FACS/CyTOF analyses. It is unclear how alternate PBMC processing protocols (e.g., RBC lysis) might change viability or population composition, which has been studied recently^18^ and could be further addressed in future studies. Targeted and unbiased analyses identified many different cellular changes across conditions, and we have made these accessible via iVET so readers can focus on their cells of interest. Cellular findings based on CyTOF data were validated with independent Astrolabe software. While observed percent changes in caspase 3 positive subfractions of cell populations were small, it is likely that small amounts of cleaved caspase 3 are sufficient to induce apoptosis and that cells with higher levels are rapidly degraded without time for cytometric detection. Future studies using controls with apoptosis inhibitors to confirm the impact cell death on population frequencies will be important to elucidate this relationship. These future studies will address whether cell subset frequency changes due to processing delay and refrigeration represent selective cell death and/or survival.

Given our focus on effects of processing delay, this study did not address other pre-analytical variables such as the effect of different anticoagulant or post-processing variables common in clinical research including freezer storage temperature and duration of plasma/serum/PBMCs, or time-delay after PBMC fixation and staining for flow/mass cytometry. Similarly, this study was not powered to assess the effects of demographic variables in our subject population. The effect of some of these variables has been reported for levels of various circulating cytokines^19^.

Experiments designed to measure immune analytes in human blood should carefully consider the impact of processing time delay and temperature. In the case of whole blood gene expression, we found a slightly reduced yield and quality of RNA from blood stored at RT. However, no significant effects were detected in pooled gene expression levels. Circulating cytokine studies should consider the non-equivalence between serum and plasma and weigh the impact of temperature and processing delay on select cytokines. Similarly, flow and mass cytometry studies should avoid time delays greater than 5 h if studying susceptible cell populations, while surprisingly temperature was not associated with as many differences in this study. Finally, we developed a web-based application tool to allow investigators to identify the effects of time and temperature during processing on stability or volatility of transcripts, cytokines, and immune cell populations of interest.

## Materials and methods

### Healthy donors, blood collection, and processing conditions

Healthy donor adults (age 18–65) were included in this study if they reported no chronic medical condition or medical therapy, and no experience of fever or recent infection (urinary, upper respiratory, gastrointestinal, etc.) within ten days of enrollment. After informed consent, peripheral venous blood was drawn between 08:00 and 09:00 by antecubital venipuncture performed by a trained phlebotomist in the Stanford Hospital Lab. Blood was drawn directly into Sodium Heparin BD Vacutainer (Ref 366480) and Serum BD Vacutainer (Ref 366430) tubes. Samples consisting of multiple tubes collected from each individual at the same time were immediately taken to the lab and processed within ten minutes of phlebotomy (baseline), or left on the bench top at room temperature (RT) for 2 h or 5 h, or refrigerated at 4 °C for 5 h or 15 h before processing. Each set of blood samples was subjected to all the processing delay conditions, so that each individual subject served as his/her own control. At baseline or after the specified processing delays, serum, plasma and PMBCs were collected by standard protocols (for details, see Supplemental Materials). This study was approved by the Stanford Institutional Review Board (IRB-42109), and all research was performed in accordance with Stanford’s Institutional Review Board guidelines and regulations, including obtaining informed consent from all participants. All blood collections, processing, data collection, and analysis were conducted between October 2017 and December 2019.

### Whole blood RNA and gene expression

At baseline or after specified processing delays, 2.5 mL of whole blood was drawn by syringe from a sodium heparin vacutainer tube and then allowed to passively fill a PAXgene blood RNA tube (Qiagen 762165), followed by refrigeration and freezing following the manufacturer’s instructions. RNA was extracted using the PAXgene Blood RNA Kit (Qiagen 762164) following the manufacturer’s instructions, including on column DNA digestion. Prior to freezing, a small aliquot was used for quality and concentration estimation using an Agilent 2100 Bioanalyzer (Stanford Protein and Nucleic Acid Facility). Sample labeling, microarray hybridization and washing were performed by Thermo Fisher Scientific (Santa Clara, CA, USA) using the Clariom D Human Assay Microarray. Arrays were scanned using a GeneChip Scanner 3000 7G (Affymetrix, Santa Clara, CA, USA), and acquired array images analyzed using Affymetrix GeneChip Operating Software. Subsequent data processing and quality control filters were performed using Transcriptome Analysis Console (TAC) Software (Thermo Fisher). Two samples, HBP-04 4C_15hr and HBP-09 4C_15hr were excluded from further analysis due to failing ThermoFisher quality control metrics with low separation between positive and negative probe controls. Statistical analysis and visualization were done using R software (R version 3.4.4). This data set can be found in GEO (GSE147788). The Clariom D Assay provides full coverage of the transcribed genome including all known coding and non-coding genes, exons, and alternative splicing variants that give rise to coding RNA and long noncoding RNA isoforms. This microarray also detects rare and low-expressing transcripts otherwise not detected by common sequencing approaches. This is why the reported number of 540,000 transcripts exceeds the known canonical gene transcripts.

### Luminex cytokine array

Cytokine measurements in plasma and serum from each participant were obtained using the Human 62-multiplex array on the Luminex 200 IS system (Affymetrix) performed at the Stanford Human Immune Monitoring Center (HIMC). The manufacturer’s protocol was followed, with variations as described by Brodin et al.^20^. Thawed plasma samples were centrifuged at maximal speed in a microcentrifuge for 10 min and supernatants entered in two replicate wells, ensuring all samples for each individual were in the same plate to avoid confounding sample processing condition with plate artifacts. Median fluorescence intensity (MFI) was preprocessed for each cytokine through a sequence of averaging over duplicate wells, natural-logarithm transformation to reduce variance heterogeneity, and isolation and removal of plate effects as previously reported^21^. Average log_2_(MFI) per condition for each cytokine was calculated, and percent change from baseline for each subject was calculated and displayed in heatmaps. Cohort variance for each processing condition was also calculated and plotted into heatmaps.

### Mass cytometry (CyTOF)

Surface and intracellular cytokine mass cytometry (CyTOF) was performed at the Stanford HIMC on viably cryopreserved PBMCs according to published methods^22^, unless otherwise noted, in batches of 20 samples per day (including all conditions for two individuals to avoid batch effects when comparing individuals to their own control conditions) with the same Helios instrument and operator. All antibody conjugates were validated for accurate detection of their respective antigens and to ensure minimal isotope spillover by the Stanford HIMC (Supplemental Table 6). Cell thawing and antibody labeling were done following standard protocols^23^ (see supplemental materials for details). Approximately 300,000 events or all possible events (whichever lower) were acquired. Bead normalized files were analyzed in FlowJo v10 for gating and concatenating sample files (Supplemental Fig. 5). Cytobank and VorTeX^24^ were used for analysis of live single cells exported from FlowJo.

### Immuno-analyte Variance Estimation Tool (iVET)

The Immuno-analyte Variance Estimation Tool (iVET) was built using the React framework^25^ and the Google Cloud product Firebase^26^, which provide a cloud database and hosting for the iVET website. All immune parameter data from the microarray, Luminex, and CyTOF assays was uploaded to the Firebase database for open online access.

### Statistical analysis

Sample size estimation of 9 to provide 80% power in a two-tailed approach was based on a recent study that measured the mean difference of a leukocyte subset percentage in anticoagulated blood samples processed immediately or processed with a 2 h delay by paired Student’s T-test^9^. Data were analyzed using R, Microsoft Excel, GraphPad Prism 7.0, and BioVinci 1.1.5. Values were considered significantly different when *p* < 0.05. Gene expression principal components analysis was calculated using the *prcomp* function in the *stats* package of R. Pearson correlations were calculated using the *cor.test* function in the *stats* package of R. For targeted analysis of the cell death pathway, TAC software (version 4.0.1) was used to identify genes whose descriptions included “cell death” or “apoptosis”. Luminex cytokine comparisons were done by paired 2-way ANOVA analysis (time vs. cytokine) with Bonferroni’s multiple comparison testing (time within each cytokine). Mass cytometry comparisons were done by paired two-tailed Student’s T-test with BH correction for multiple comparisons when indicated.

For CyTOF analysis using Astrolabe, single-cell data was clustered using the FlowSOM R package^27^ and labeled using the Ek'Balam algorithm^13^. Differential abundance analysis was done using the edgeR R package^28,29^ following the method outlined in Lun et al.^30^. Cell subset definitions follow Maecker et al.^31^, and Finak et al.^32^. Cluster labeling, method implementation, and visualization were done through the Astrolabe Cytometry Platform (Astrolabe Diagnostics, Inc.).

## Supplementary information

Supplementary Information 1.

Supplementary Figures.

Supplementary Information 2.

## Abbreviations

PBMC: Peripheral blood mononuclear cells
RT: Room temperature
