# Supplementary material for: Effects of processing conditions on stability of immune analytes in human blood
Source: Sci Rep. 2020 Oct 15;10:17328. doi: 10.1038/s41598-020-74274-8 (PMC7566484; doi:10.1038/s41598-020-74274-8)
Supplement: Supplementary file 1 — Supplementary Information 1. [file 41598_2020_74274_MOESM1_ESM.pdf]

TITLE: Effects of processing conditions on stability of immune analytes in human blood

Short Title: Blood processing delay effects on immune analytes

Authors: Andres Gottfried-Blackmore, MD PhD[1#*]; Samuel J.S. Rubin, PhD[2#]; Lawrence Bai, BS[2#]; Sheun Aluko, BS[3], Yujun Yang, MD[1]; Walter Park[1], MD; Aida Habtezion, MD MSc[1*].

Institution: 1. Division of Gastroenterology and Hepatology, Department of Medicine, Stanford University School of Medicine, Alway Building M211, 300 Pasteur Drive, Stanford, CA, 94305, USA. 2. Immunology Program, Stanford University School of Medicine, 1215 Welch Road, Modular B, Stanford, CA, 94305, USA. 3. Biomedical Informatics, Stanford University School of Medicine, 291 Campus Drive, Li Ka Shing Building, Stanford, CA 94305

*Corresponding Authors:
Andres Gottfried-Blackmore (ORCID 0000-0002-3563-3727) & Aida Habtezion (ORCID 0000-0002-4473-006X)
300 Pasteur Dr. Alway Bld. M211, Stanford, CA 94062, USA
Email: andresg@stanford.edu; aidah@stanford.edu
Fax. 01-650-723-5488; Tel.01-650-7235512

# equal contribution.

Abbreviations:
PBMC: peripheral blood mononuclear cells; RT: room temperature

Supplemental Methods:

Preparation and Storage of Serum, Plasma, and Peripheral Blood Mononuclear Cell suspensions: At baseline or after the specified processing delays serum vacutainer tubes were centrifuged at 1200 rcf for 10 minutes at room temperature; serum supernatant was aliquoted and frozen at -80°C. Plasma was generated by centrifugation of sodium heparin vacutainer tubes at 800 rcf for 10 minutes at room temperature; plasma supernatant was aliquoted with care not to pipette any cells and frozen at -80°C. The remaining blood in the plasma tubes was then diluted 1:1 with PBS without calcium or magnesium, overlaid onto 15 mL of Ficoll-Paque (GE Healthcare, cat. #17-1440-03) in Accuspin tubes (Sigma-Aldrich, cat. #A2055), and centrifuged at 800 rcf for 20 minutes at room temperature with acceleration at five and break at zero. The buffy coat PBMC layer was collected and washed twice in 50 mL PBS by centrifugation at 800 rcf for 10 minutes at RT. PBMCs were resuspended in Recovery Cell Culture Freezing Medium (Thermo Fisher Scientific, cat. #12648010) at 5-10x10^6 cells/ 1 mL and frozen according to manufacturer instructions (Bel-Art Products, cat. #F18844-0000), and then stored in liquid nitrogen.

Mass Cytometry (CyTOF) cell preparation and labeling

Briefly, PBMCs were thawed in warm media (RPMI containing 10% FCS and 1% penicillin/streptomycin) with benzonase (25 U/ml), washed twice in media with benzonase, washed once in media without benzonase, and 1 million cells were rested in media without benzonase in a 96-well deep well plate for 2-3 h at 37ºC before stimulation with PMA (10ng/ml), ionomycin (1μg/ml), and LPS (1μg/ml) in the presence of brefeldin A (5μg/ml) and monensin (5μg/ml) for 4 h. An unstimulated control sample was prepared for each subject/condition and treated the same as stimulated samples, except for addition of stimulants. Anti-CD107a and –CD154 antibodies (2μg/ml) were added during stimulation according to referenced methods.

EDTA was added (2mM) for 15 min at room temperature, before washing twice with CyFACS buffer (CyPBS (Rockland) containing BSA (0.1%), EDTA (2mM) and sodium azide (0.05%)). Cells were incubated with a 70µL cocktail of antibodies against cell surface antigens (Supplemental Table 1) in CyFACS buffer for 45 min on ice, washed twice with CyFACS buffer and resuspended in CyPBS (100µL) containing of 1:3000 maleimide-DOTA and 115-Indium for 30 min on ice. Cells were washed thrice in CyFACS buffer, resuspended in CyPBS (100µL) containing 2% PFA and incubated overnight at 4ºC. After washing in 1X permeabilization buffer (eBioscience), cells were incubated with a 70µL cocktail of antibodies against intracellular antigens (Supplemental Table 1) in CyFACS buffer for 45 min on ice. After washing thrice in CyFACS buffer, cells were incubated in CyPBS (100µL) containing 1:2000 Ir-Intercalator and PFA (2%) for 20 min at RT, washed twice in CyFACS followed by milliQ water thrice, spiked with beads according to the manufacturer's instructions, and resuspended in 1-1.5mL milliQ water for analysis on a Helios instrument. Approximately 300,000 events or all possible events (whichever lower) were acquired for each sample. Bead normalized files were obtained from the Helios instrument using on-board software.

Immune-analyte Variance Estimation Tool (iVET) web application

Overview

The iVET web application was created using the Google application development software suite known as *Firebase* (https://firebase.google.com/*),* which provides all necessary components for building a modern cloud hosted website that is accessible anywhere in the world.

The software architecture consists of 2 main components:

1. A cloud hosted database called *Firestore*, which stores all of the immuno-analyte data.
2. The website interface which was programmed using the React javascript framework, developed by Facebook for programming websites (https://reactjs.org/).

When a user visits the iVET website URL, the website code is delivered to their web browser in order to run the iVET application. When the user searches for an analyte of interest, iVET queries the *Firestore* database over the internet in order to retrieve the specific immune analyte data requested, and then graphs the data.

Cloud Database and Data Format

The immune analyte data is stored in a cloud hosted database called *Firestore.* Firestore is a *document* database as opposed to a relational (SQL, or server query language) database, with the main difference being that a document database has a flexible and updateable schema (data model), while a SQL database has a fixed data model (https://aws.amazon.com/nosql/document/). In a document database, the data is partitioned into *collections,* each of which consists of an arbitrarily large number of *documents.* Each document is a *key-value* store, which is a data structure that maps specific keys to their corresponding values. For the purposes of iVET, each *collection* corresponds to a separate assay and/or sub condition for that assay. For example, “stimulated CyTOF”, “unstimulated CyTOF” and “microarray” are collections. Each collection contains several “documents”, where each document holds all of the data for a specific analyte within that collection. For example, within the “plasma cytokines” collection, the document “IL1A” houses all of the IL1A measurements for all 10 patients across all time and temperature conditions. The key value format of each document is consistent across all collections. Namely, the keys consist of the the patient and time/temperature identifier (for example, “HBP01-1”, which identifies patient 1 and time/temperature condition 1). The corresponding value is the numeric quantity associated with that patient, at that condition, for the immune analyte that corresponds to the document.

Website Interface

The iVET interface was created using the React Javascript Framework. Specifically, the *create-react-app* (https://github.com/facebook/create-react-app) command line tool was used to

initiate the project source code, and this base code was heavily modified and adapted to create the desired interface.

The interface consists of 3 main components:

a. Assay selection dropdown
b. Analyte search box
c. Data format selector
d. Box plot data visualization

React javascript serves as the backbone programming paradigm for the web application. However, in modern web development a “component library” is often chosen for styling applications and for providing modular functionality, such as search bars, dropdown menus, etc. iVET utilizes the *semantic-ui* component library (https://react.semantic-ui.com/) for components a, b, and c above. The lists of selectable analytes for each assay is stored within the iVET interface. Thus, when a user selects a specific assay using the dropdown menu, the search bar component is limited to only display candidate results from that assay. This programmability is achieved using javascript. When a user selects a particular assay and analyte (IL1A, for example), the corresponding *document* is retrieved from the database using a javascript HTTP request and the raw values are graphed directly by iVET. Specifically, all of the values across 10 patients for time/temperature condition 1 are pooled together to produce the first box plot. A similar process is performed to produce the remaining 4 box plots corresponding to the other 4 time/temperature conditions. The only mathematical computation coded in the iVET application is the calculation of the percentage change. This is done by repeating the following calculation for *each* patient: for N=2..4, we calculate ((the patient’s value for the assay at time/temperature condition N) - the patient’s baseline value) / (the patient’s baseline value), where *baseline value* is simply the value for the assay at time/temperature condition 1. After this calculation is performed, iVET displays these percentage changes pooled together across patients for each N, producing the N box plots. Both the raw values and the percent change values are sent to

the graphs; however, only one is plotted at a time. When the user toggles the data format to percent change or raw values, a message is sent to the box plot to update the graph to show the desired format.

The box plots are created using the open source web plotting library *Plotly* (*https://plotly.com/javascript/*), which was integrated into iVET. Once the data values are retrieved as described above, they are passed to the graphing library as 5 separate arrays, where each array represents a time/temperature condition, and the values in each array correspond to the immune anayte values for each patient for that condition. *Plotly* automatically plots median, minimum, maximum, and quartile values based on the input data.

Supplemental Figures:

Supplemental Figure 1. **Prolonged time delay has minimal impact on RNA yield and RIN values.** Bar graphs showing (A) RNA concentration, (B) % of samples with RIN≥8, and (C) the RNA QC metric based on microarray gene expression profiles of positive vs negative AUC values. The pos vs neg AUC for HBP04-5 and HBP09-5 were evaluated as low and further excluded from the study (see Methods).

Supplemental Figure 2. **PCA analysis does not reveal any separation by variance across independent demographic variables such as age, sex, race, and individual subjects.** PC analysis of samples containing all conditions, colored by (A) age; (B) sex; (C) race; and (D) individual subject.

Supplemental Figure 3. **Plasma and serum cytokine levels are poorly correlated**. Heatmap showing Pearson correlation coefficients between serum and plasma cytokine levels (log2 MFI), ranked at baseline, and shown for all four processing delay conditions. (* = p<0.05; repeated-measures Two-Way ANOVA Bonferroni posthoc test for multiple comparisons).

Supplemental Figure 4. **Processing delay exerts relatively greater changes in circulating cytokines as a function of group median**. Heatmaps showing % change in 62 cytokines from median group values at baseline. (A) Serum and (B) plasma cytokines grouped by putative functional groups and ranked by abundance in blood for each subgroup. (C) PCA plots showing effect of temperature (color code) and time (circle size) for each individual on aggregate cytokine variation in serum and plasma. (* = $p<0.05$, ** = $p<0.01$, *** = $p<0.001$; repeated-measures Two-Way ANOVA Bonferroni posthoc test for multiple comparisons).

Supplemental Figure 5. **CyTOF gating schemes**. (A) Gating scheme used to identify canonical cell populations for a representative sample. (B) Gating scheme used in viSNE analysis from Supplemental Figure 10 for a representative sample. (C) Representative viSNE plots for each condition colored by overlaid IL-2 expression used for manual screening as described in Supplemental Figure 10A. Red arrows, visual differences from baseline.

Supplemental Figure 6. **CyTOF viSNE analysis identifies significant differences between baseline and 15 h 4°C conditions**. Using viSNE, we clustered cells from all individuals concatenated by condition in an unsupervised manner and then compared gross differences in overlaid marker expression. Unsupervised clustering was used for individual samples and then models were constructed by the algorithm to distinguish baseline and 15 h conditions. Based on trends identified visually from viSNE, we manually gated cell frequencies and median signal intensities for each trend and tested these for statistical significance. (A) viSNE was run on live singlets concatenated by sample group for unstimulated or stimulated samples (10 groups), where each concatenated file was randomly down sampled to 100,000 events. The algorithm was run with 5000 iterations, perplexity 75, theta 0.5, and clustering channels CD16, CD69, TCRgd, CD27, CD154, CD107a, CD45RA, CD38, CD25, CD33, CD14, CD127, CD19, CD4, CD8, CD40, HLA-DR, CD20, CD3, CCR7, CD21, CD56. Trends were identified visually from

viSNE overlay plots (Supplemental Figure 6B-C), gated manually, and tested for statistical significance using raw values (representative example shown). (B) All significant differences identified from visual trends tested. Mean % change was calculated by dividing the latter time point by the prior time point for each respective individual, multiplying by 100, subtracting 100 to get the % change, and then computing the mean of each individual's % change. Statistics: paired two-tailed Student's T-test. *, largely driven by one outlier with few CD33+ cells at 0 h; mean % change = -33.10 without this individual.

Supplemental Figure 7. **CyTOF CITRUS analysis identifies significant differences between baseline and 15 h 4°C conditions**. Using CITRUS, we conducted supervised comparisons between baseline and 15 h conditions as above, since all viSNE and most other trends were identified between baseline vs. 15 h conditions. Unsupervised clustering was used for individual samples and then models were constructed by the algorithm to distinguish baseline and 15 h conditions. Based on trends identified visually from viSNE and computationally from CITRUS, we manually gated cell frequencies and median signal intensities for each trend and tested these for statistical significance. (A) CITRUS was run on live singlets for each sample. X-Shift/VorTeX was used to determine the optimal cluster number (87), minimum cluster size was set accordingly, and each sample file was randomly down sampled to 10,000 events. The algorithm was used to compare unstimulated or stimulated baseline vs. 15 h conditions using with 5 cross validation folds, 1% false discovery rate, normalized scales, and clustering channels CD16, CD69, TCRgd, CD27, CD154, CD107a, CD45RA, CD38, CD25, CD33, CD14, CD127, CD19, CD4, CD8, CD40, HLA-DR, CD20, CD3, CCR7, CD21, CD56. Trends identified by CITRUS were gated manually and tested for statistical significance using raw values (representative example shown). (B) All significant differences identified from CITRUS trends tested. Mean % change was calculated by dividing the latter time point by the prior time point for

each respective individual, multiplying by 100, subtracting 100 to get the % change, and then computing the mean of each individual's % change. Statistics: paired two-tailed Student's T-test.

Supplemental Figure 8. **Independent analysis of CyTOF data with Astrolabe is consistent with other guided analysis approaches**. Astrolabe is an independent platform that allows standardized, literature-based analysis of cytometry data, including preprocessing, clustering, cell subset labeling, and differential analysis of sample conditions based on cell subset abundance and marker expression. Raw CyTOF data FCS files were uploaded to the Astrolabe platform for independent analysis to validate findings from previous guided analyses. Astrolabe software performed all data preprocessing, unsupervised clustering (left plot), cell subset labeling, and differential analysis between sample conditions for cell subset abundance and marker expression (center and right plots). For comparisons between five conditions, differentially represented cell subsets were largely consistent with other guided analyses. For example, (A) naive CD8+ T cell frequency (green dot, center volcano plot) as % of live singlets was decreased in the 15 h 4ºC condition for unstimulated samples (right plot), and (B) CD14+CD16- monocyte frequency (green dot, center volcano plot) as % of live singlets was decreased in the 5 h and 15 h 4ºC conditions for stimulated samples (right plot). Statistics: edgeR R package using Astrolabe (see Methods).

Supplemental Tables:

Supplemental Table 1. Kruskal-Wallis test with multiple comparisons across the five delay conditions revealed no statistically significant gene expression difference.

(See attached cvs spreadsheet)

Kruskal-Wallis test was done for each gene measured across the four groups (A-D; 210 genes total) and p-values were adjusted using the Benjamini-Hochberg method (BH). Genes were considered significant if adjusted p-values were < 0.05.

Supplemental Table 2. **Many of the 284 gated cell parameters were differentially preserved after blood draw**.

Condition 1	Condition 2	Population	P-value	Mean % change (condition 1 to 2)
5hr RT UNSTIM	5hr 4C UNSTIM	CD45RA+CCR7+ (% of CD8+ T cells)	0.0234	-74.32
0hr RT UNSTIM	5hr RT UNSTIM	Perforin+ (% of CD27+CD20+ B cells)	0.0397	-22.51
0hr RT UNSTIM	15hr 4C UNSTIM	Lymphocytes (% of live singlets)	0.0008	13.50
		CD45RA+CCR7+ (% of CD8+ T cells)	0.0365	-36.45
		Monocytes (% of live singlets)	0.0007	-69.43
0hr RT STIM	15hr 4C STIM	Lymphocytes (% of live singlets)	0.0060	9.20
		CD8+ (% of T cells)	0.0462	-22.24
		CD107a+ (% of CD8+ T cells)	0.0002	51.24
		IFNg+ (% of CD8+ T cells)	0.0130	72.83
		MIP1b+ (% of CD8+ T cells)	0.0407	58.59
		IL2+ (% of CD45RA-CCR7+CD8+ T cells)	0.0422	78.75
		IL4+ (% of CD45RA-CCR7+CD8+ T cells)	0.0472	115.02
		TNFa+ (% of CD45RA-CCR7+CD8+ T cells)	0.0104	51.24

		IL2+ (% of CD45RA+CCR7+CD8+ T cells)	0.0068	86.25
		TNFa+ (% of CD8+ T cells)	0.0139	72.58
		GMCSF+ (% of NKT cells)	0.0371	98.13
		IFNg+ (% of CD16lo NK cells)	0.0279	49.57
		TNFa+ (% of CD16lo NK cells)	0.0006	69.40
		Monocytes (% of live singlets)	0.0223	-81.08

284 cell population frequencies were gated between five conditions for unstimulated and stimulated samples, and significant differences are shown. Each condition was compared to baseline for unstimulated or stimulated samples, and significant differences were identified using raw values. Room temperature was also compared to 4°C for the 5 h conditions. Mean % change was calculated by dividing the latter or colder time point by the prior or room temperature time point for each respective individual, multiplying by 100, subtracting 100 to get the % change, and then computing the mean of each individual's % change. Statistics: paired two-tailed Student's T-test with BH method correction for multiple comparisons.

Supplemental Table 3. **Cytokine-positive cell frequencies for IL2, TNFα, and IFNγ are differentially preserved after blood draw**.

Condition 1	Condition 2	Population	P-value	Mean % change (condition 1 to 2)
5hr RT STIM	5hr 4C STIM	TNFa+ (% of CD16- monocytes)	0.0187	-41.43
0hr RT STIM	5hr 4C STIM	IFNg+ (% of CD16lo NK cells)	0.0320	39.34
0hr RT STIM	15hr 4C STIM	IFNg+IL2+TNFa+ (% of CD4+ T cells)	0.0408	113.17
		IFNg+ (% of CD8+ T cells)	0.0026	72.83
		IL2+ (% of CD8+ T cells)	0.0200	63.25
		IFNg+ (% of CD45RA-CCR7+CD8+ T cells)	0.0170	40.07
		IL2+ (% of CD45RA-CCR7+CD8+ T cells)	0.0084	78.75

		TNFa+ (% of CD45RA-CCR7+CD8+ T cells)	0.0021	51.24
		IL2+ (% of CD45RA+CCR7+CD8+ T cells)	0.0014	86.25
		IL2+ (% of CD45RA+CCR7-CD8+ T cells)	0.0473	102.97
		TNFa+ (% of CD45RA+CCR7-CD8+ T cells)	0.0111	19.34
		IL2+ (% of CD45RA-CCR7-CD8+ T cells)	0.0128	86.82
		TNFa+ (% of CD45RA-CCR7-CD8+ T cells)	0.0113	33.70
		TNFa+ (% of CD8+ T cells)	0.0027	72.58
		IFNg+ (% of gdT cells)	0.0144	39.48
		TNFa+ (% of gdT cells)	0.0478	33.33
		IFNg+ (% of NKT cells)	0.0429	46.89
		IL2+ (% of NKT cells)	0.0333	136.62
		TNFa+ (% of NKT cells)	0.0146	32.81
		IFNg+ (% of CD16lo NK cells)	0.0056	49.57
		TNFa+ (% of CD16lo NK cells)	0.0001	69.40

A total of 64 IL2-, TNFα-, and IFNγ-positive frequencies were gated for canonical cytokine-producing cell populations between five conditions for unstimulated and stimulated samples, and significant differences based on raw values are shown. Each condition was compared to baseline for unstimulated or stimulated samples, and significant differences were identified. Room temperature was also compared to 4°C for the 5 h conditions. Mean % change was calculated by dividing the latter or colder time point by the prior or room temperature time point for each respective individual, multiplying by 100, subtracting 100 to get the % change, and then computing the mean of each individual's % change. Statistics: paired two-tailed Student's T-test with BH method correction for multiple comparisons.

Supplementary Table 4. **Several of 365 calculated cell population median signal intensities are differentially preserved after blood draw**.

Condition 1	Condition 2	Median marker (cell population)	P-value	Mean % change (condition 1 to 2)
5hr RT UNSTIM	5hr 4C UNSTIM	CD25 (CD45RA-CCR7+CD4+ T cells)	0.0401	-66.70
0hr RT UNSTIM	5hr RT UNSTIM	Perforin (CD45RA-CCR7+CD4+ T cells)	0.0292	-43.32
0hr RT UNSTIM	15hr 4C UNSTIM	TNFα (CD45RA+CCR7-CD8+ T cells)	0.0197	-46.38
		IL-4 (HLA-DR+CD38+CD8+ T cells)	0.0039	-66.03
		CTLA-4 (HLA-DR+CD38+CD8+ T cells)	0.0070	-43.29
0hr RT STIM	15hr 4C STIM	CTLA-4 (CD45RA-CCR7-CD8+ T cells)	0.0353	-56.51
		IFNγ (NKT cells)	0.0134	1822.43
		CD69 (CD27+CD20+ B cells)	0.0382	-56.23
		CTLA-4 (CD16hi NK cells)	0.0161	-55.70
		CTLA-4 (CD16lo NK cells)	0.0054	-59.76
		CTLA-4 (CD16+ monocytes)	0.0279	-53.21

365 cell population median signal intensities were calculated between five conditions for unstimulated and stimulated samples, and significant differences are shown. Each condition was compared to baseline for unstimulated or stimulated samples, and significant differences were identified using raw values. Room temperature was also compared to 4C for the 5 hour conditions. Mean % change was calculated by dividing the latter or colder time point by the prior or room temperature time point for each respective individual, multiplying by 100, subtracting 100 to get the % change, and then computing the mean of each individual's % change. Statistics: paired two-tailed Student's T-test with BH method correction for multiple comparisons.

Supplemental Table 5. **CyTOF analysis of Caspase 3 in canonical cell populations**.

Condition 1	Condition 2	Population	P-value	Mean % change (condition 1 to 2)

0hr RT UNSTIM	2hr RT UNSTIM	Caspase 3+ (% of CD45RA-CCR7- CD4+ T cells)	0.0262	-6.60
		Caspase 3+ (% of CD8+ T cells)	0.0226	-27.62
0hr RT UNSTIM	5hr RT UNSTIM	Caspase 3+ (% of live singlets)	0.0272	-29.91
		Caspase 3+ (% of CD4+ T cells)	0.0009	-39.98
		Caspase 3+ (% of CD45RA-CCR7+ CD4+ T cells)	0.0031	-42.09
		Caspase 3+ (% of CD45RA+CCR7+ CD4+ T cells)	0.0265	-41.30
		Caspase 3+ (% of CD45RA-CCR7- CD4+ T cells)	0.0358	-33.18
		Caspase 3+ (% of CD8+ T cells)	0.0051	-39.93
		Caspase 3+ (% of CD45RA-CCR7+ CD8+ T cells)	0.0077	-43.69
		Caspase 3+ (% of CD45RA+CCR7+ CD8+ T cells)	0.0168	-49.48
0hr RT UNSTIM	5hr 4C UNSTIM	Caspase 3+ (% of lymphocytes)	0.0071	1.44
		Caspase 3+ (% of CD3+ cells)	0.0264	1.43
5hr RT STIM	5hr 4C STIM	Caspase 3+ (% of lymphocytes)	0.0048	62.65
0hr RT STIM	5hr RT STIM	Caspase 3+ (% of CD45RA-CCR7+ CD8+ T cells)	0.0340	-47.83
0hr RT STIM	15hr 4C STIM	Caspase 3+ (% of CD16lo NK cells)	0.0297	-43.31

Caspase 3-positive frequencies were gated for 26 canonical cell populations between five conditions for unstimulated and stimulated samples, and significant differences based on raw values are shown. Each condition was compared to baseline for unstimulated or stimulated samples, and significant differences were identified. Room temperature was also compared to 4°C for the 5 h conditions. Mean % change was calculated by dividing the latter or colder time point by the prior or room temperature time point for each respective individual, multiplying by 100, subtracting 100 to get the % change, and then computing the mean of each individual's % change. Statistics: paired two-tailed Student's T-test with BH method correction for multiple comparisons.

Supplemental Table 6. CyTOF antibody panel.

Metal	Marker	Staining	Clone	Source	Catalog No.
113In	CD57	Surface	HCD57	BioLegend/Stanford HIMC	Custom
115In	Live/dead	Intracell	n/a	Stanford HIMC	n/a
140Ce	Beads	n/a	n/a	Fluidigm	201078
141Pr	CD25	Surface	3C7	BioLegend/Stanford HIMC	10193
142Nd	CD19	Surface	HIB19	Fluidigm	3142001B
143Nd	IL-10	Intracell	JES3-9D7	BioLegend/Stanford HIMC	501423
144Nd	IL-4	Intracell	MP4-25D2	Fluidigm	3144010B
145Nd	CD4	Surface	RPA-T4	Fluidigm	3145001B
146Nd	CD8	Surface	RPA-T8	Fluidigm	3146001B
147Sm	CD20	Surface	2H7	Fluidigm	3147001B
148Nd	CD40	Surface	5C3	BioLegend/Stanford HIMC	334325
149Sm	CTLA-4	Surface	14D3	eBioscience/Stanford HIMC	14-1529-82
150Nd	MIP1b	Intracell	D21-1351	Fluidigm	3150004B
151Eu	CD107a	Surface*	H4A3	Fluidigm	3151002B
152Sm	TNFα	Intracell	Mab11	Fluidigm	3152002B
153Eu	CD45RA	Surface	HI100	Fluidigm	3153001B
154Sm	CD3	Surface	UCHT1	Fluidigm	3154003B
155Gd	CD36	Surface	5-271	Fluidigm	3155012B
156Gd	CD38	Surface	HB-7	BD Biosciences/Stanford HIMC	347680
157Gd	HLA-DR	Surface	G46-6	BD Biosciences/Stanford HIMC	556642
158Gd	CD33	Surface	WM53	Fluidigm	3158001B
159Tb	GMCSF	Intracell	BVD2-21C11	Fluidigm	3159008B
160Gd	CD14	Surface	M5E2	Fluidigm	3160001B
161Dy	IFNγ	Intracell	4S.B3	eBioscience/Stanford HIMC	554549
162Dy	CD69	Surface	MCA 1442	Fluidigm	Custom
163Dy	TCR$\gamma\delta$	Surface	B1	BioLegend/Stanford HIMC	331202
164Dy	IL-17	Intracell	N49-853	Fluidigm	3164002B
165Ho	CD127	Surface	A019D5	Fluidigm	3165008B
166Er	IL-2	Intracell	MQ1-17h12	Fluidigm	3166002B
167Er	CD27	Surface	L128	Fluidigm	3167006B
168Er	CD154(CD40L)	Surface*/Intracell	24-31	Fluidigm	3168006B
169Tm	CCR7	Surface	150503	R&D Systems/Stanford HIMC	MAB197-100
170Er	PD1	Surface	EH12.1	BD Biosciences/Stanford HIMC	562138
171Yb	Granzyme B	Intracell	GB11	Fluidigm	3171002B
172Yb	Caspase-3 (cleaved)	Intracell	5A1E	Fluidigm	3172023A
173Yb	Perforin	Intracell	B-D48	Abcam/Stanford HIMC	ab47225
174Yb	CD21	Surface	Bu32	BioLegend/Stanford HIMC	354902
175Lu	PD-L1	Surface	29E.2A3	Fluidigm	3175017B
176Yb	CD56	Surface	NCAM16.2	Fluidigm	3176008B

209Bi	CD16	Surface	3G8	Fluidigm	3209002B
191Ir	DNA	Intracell	n/a	Fluidigm	201192B
193Ir	DNA	Intracell	n/a	Fluidigm	201192B

* Added during stimulation.
