# Supplementary material for: Effects of processing conditions on stability of immune analytes in human blood
Source: Sci Rep. 2020 Oct 15;10:17328. doi: 10.1038/s41598-020-74274-8 (PMC7566484; doi:10.1038/s41598-020-74274-8)

**Supplemental Figure 1. Prolonged time delay has minimal impact on RNA yield and RIN values.**

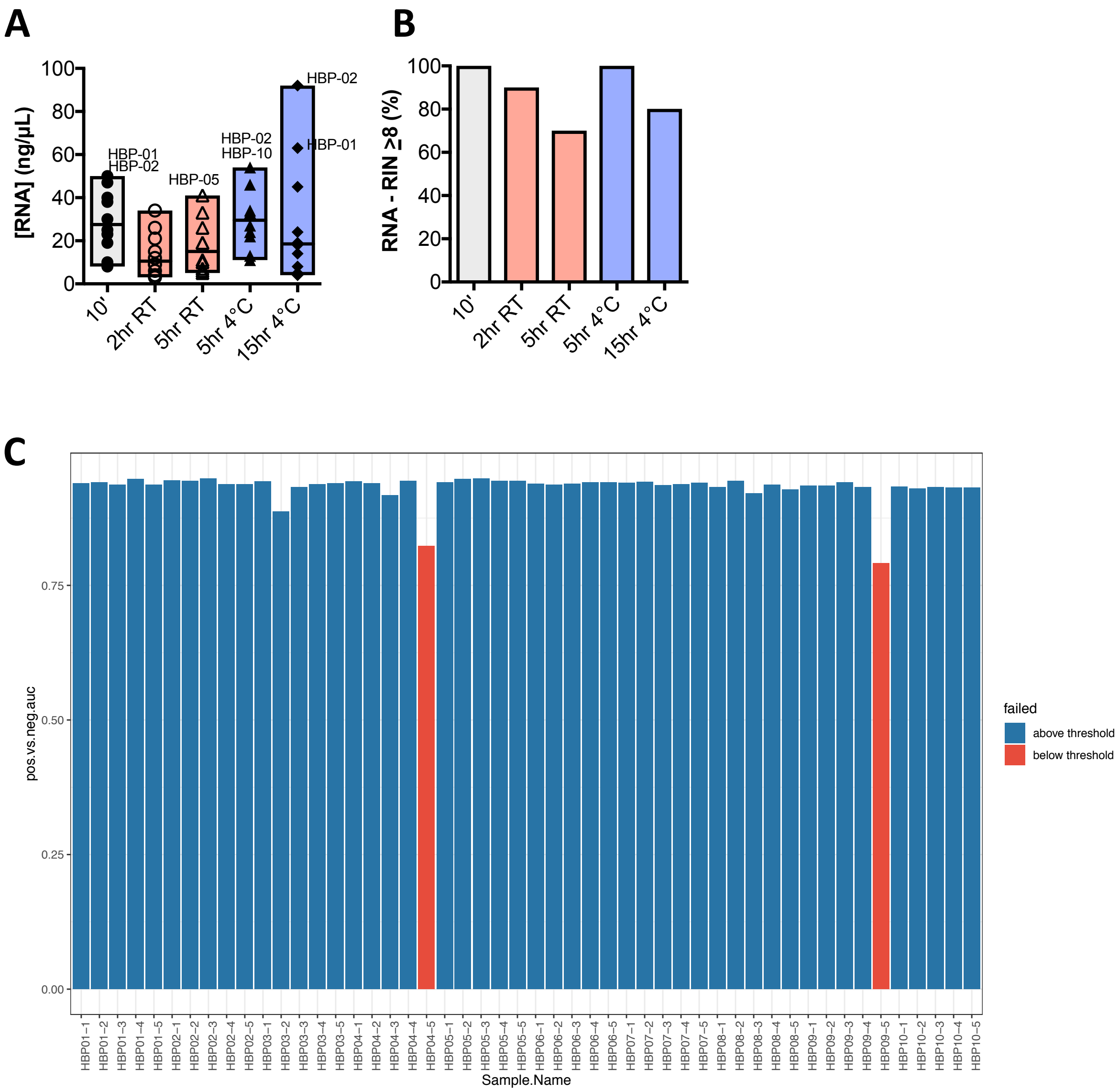

**Supplemental Figure 2. PCA analysis does not reveal any separation by variance across independent demographic variables such as age, sex, race, and individual subjects.**

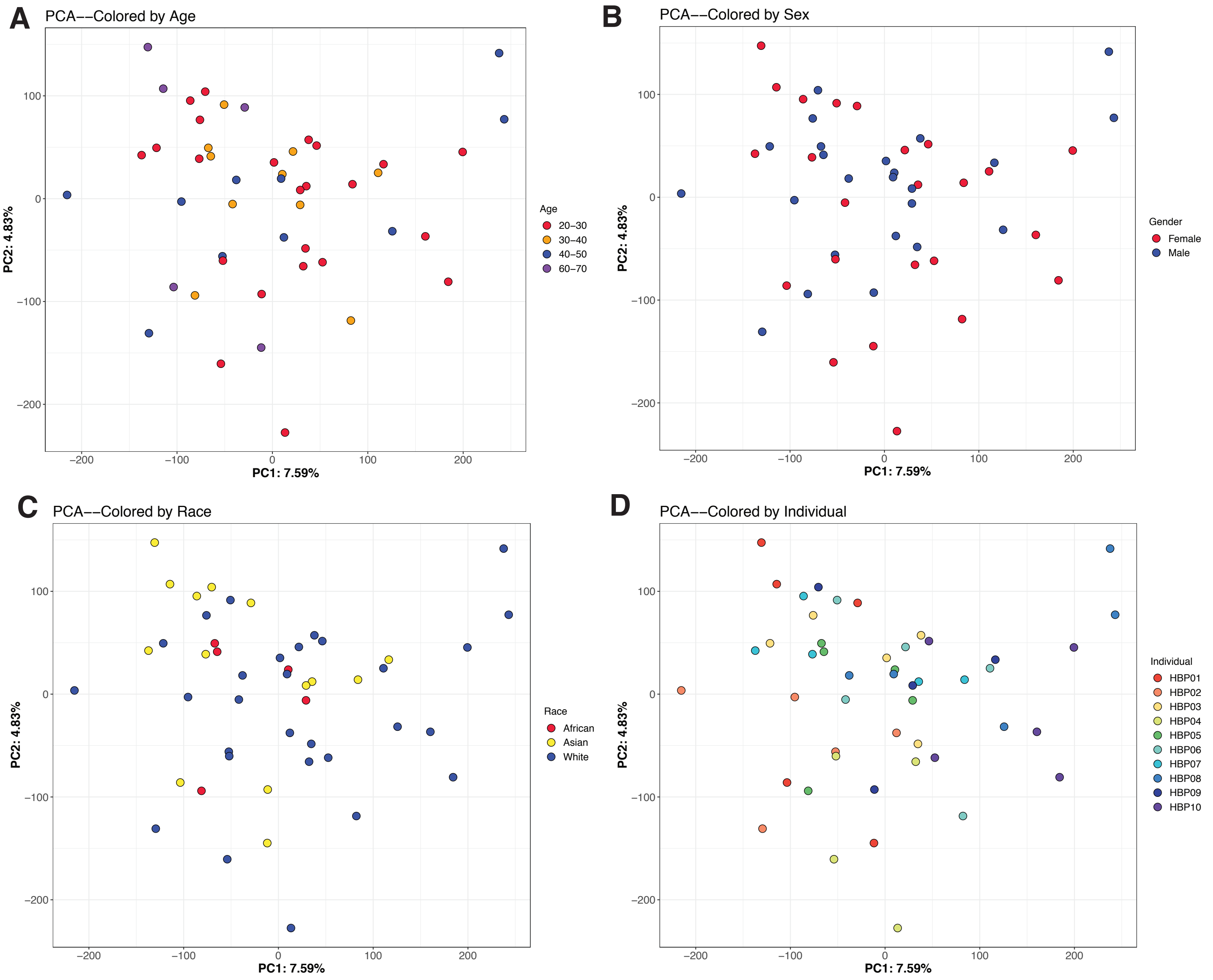

## Supplemental Figure 3. Plasma and Serum cytokine levels are poorly correlated.

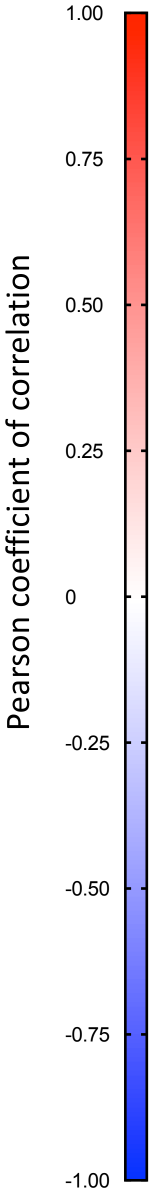


| | Baseline | 2HR $^{RT}$ | 5HR $^{RT}$ | 5HR $^{4C}$ | 15HR $^{4C}$ |
|---|---|---|---|---|---|
| MIP1A | 0.65 * | 0.14 | 0.14 | 0.14 | 0.44 |
| TGFA | 0.52 | -0.16 | 0.12 | 0.32 | -0.35 |
| SDF1A | 0.41 | 0.37 | 0.15 | 0.07 | 0.09 |
| MIP1B | 0.33 | 0.12 | -0.12 | -0.04 | 0.49 |
| IL7 | 0.25 | 0.00 | -0.18 | 0.43 | -0.03 |
| IL2 | 0.24 | 0.01 | 0.00 | 0.37 | 0.02 |
| IL15 | 0.15 | 0.05 | 0.34 | 0.52 | -0.12 |
| FASL | 0.14 | 0.21 | -0.16 | 0.32 | 0.11 |
| IP10 | 0.12 | -0.39 | 0.32 | -0.12 | -0.23 |
| EOTAXIN | 0.09 | 0.05 | 0.12 | 0.14 | -0.14 |
| IL5 | 0.09 | -0.33 | -0.25 | -0.03 | -0.15 |
| SCF | 0.09 | -0.18 | -0.27 | 0.24 | -0.06 |
| IL18 | 0.07 | -0.03 | 0.18 | 0.48 | 0.16 |
| IL23 | 0.05 | -0.25 | 0.25 | 0.14 | -0.06 |
| LIF | 0.04 | -0.12 | -0.33 | 0.51 | -0.29 |
| IFNA | 0.04 | -0.05 | 0.28 | -0.02 | 0.05 |
| PAI1 | 0.04 | 0.42 | 0.22 | 0.02 | -0.13 |
| RESISTIN | 0.03 | -0.04 | -0.04 | -0.10 | 0.11 |
| IL1A | 0.02 | 0.03 | -0.06 | 0.27 | 0.31 |
| LEPTIN | 0.02 | 0.02 | -0.07 | -0.18 | -0.17 |
| TNFB | 0.01 | 0.12 | 0.40 | 0.04 | 0.20 |
| IL13 | -0.02 | 0.13 | 0.44 | -0.19 | 0.12 |
| MIG | -0.02 | 0.00 | -0.05 | 0.11 | 0.15 |
| EGF | -0.02 | -0.32 | -0.19 | 0.27 | 0.46 |
| VEGFD | -0.02 | -0.13 | 0.04 | -0.31 | -0.17 |
| VEGF | -0.04 | -0.10 | 0.08 | -0.08 | -0.39 |
| IL1RA | -0.05 | -0.01 | 0.24 | 0.38 | 0.06 |
| NGF | -0.05 | -0.04 | 0.03 | 0.33 | 0.01 |
| IL17F | -0.07 | -0.01 | 0.71 * | -0.02 | -0.19 |
| TGFB | -0.08 | 0.04 | 0.17 | 0.04 | -0.15 |
| TRAIL | -0.08 | -0.12 | -0.17 | 0.18 | 0.10 |
| IL6 | -0.10 | -0.11 | 0.10 | 0.09 | 0.02 |
| IL10 | -0.12 | -0.10 | 0.44 | -0.01 | 0.08 |
| MCSF | -0.13 | -0.12 | 0.25 | -0.37 | -0.17 |
| MCP3 | -0.13 | -0.06 | -0.01 | 0.20 | -0.17 |
| FGFB | -0.14 | -0.23 | 0.46 | -0.36 | -0.01 |
| IL9 | -0.15 | 0.03 | -0.41 | -0.27 | 0.15 |
| IL31 | -0.15 | -0.15 | 0.20 | -0.10 | -0.01 |
| MCP1 | -0.15 | -0.19 | 0.33 | -0.15 | -0.03 |
| GROA | -0.18 | -0.14 | 0.17 | -0.11 | 0.03 |
| IL4 | -0.18 | -0.03 | -0.28 | 0.02 | -0.02 |
| HGF | -0.18 | -0.16 | 0.28 | -0.22 | -0.20 |
| IL8 | -0.20 | -0.06 | -0.64 * | 0.51 | -0.41 |
| PDGFBB | -0.21 | -0.33 | 0.36 | -0.21 | 0.03 |
| TNFA | -0.23 | 0.22 | 0.76 * | -0.35 | 0.39 |
| IL1B | -0.23 | 0.13 | 0.03 | 0.01 | -0.12 |
| IL27 | -0.23 | 0.01 | 0.07 | 0.23 | 0.11 |
| RANTES | -0.24 | -0.03 | -0.28 | -0.11 | 0.47 |
| IL21 | -0.25 | -0.10 | -0.18 | 0.20 | -0.19 |
| VCAM1 | -0.27 | 0.16 | -0.05 | 0.01 | 0.02 |
| IL12P70 | -0.28 | -0.30 | 0.35 | 0.19 | -0.11 |
| ENA78 | -0.29 | 0.43 | -0.36 | -0.32 | 0.26 |
| IL17A | -0.29 | -0.03 | 0.27 | 0.14 | 0.27 |
| IFNG | -0.35 | 0.05 | 0.40 | -0.06 | -0.13 |
| GMCSF | -0.39 | -0.11 | -0.02 | -0.08 | -0.07 |
| IFNB | -0.39 | 0.38 | -0.02 | 0.19 | -0.06 |
| IL12P40 | -0.39 | -0.20 | 0.06 | -0.21 | 0.21 |
| CD40L | -0.43 | -0.34 | -0.30 | -0.07 | -0.06 |
| IL22 | -0.43 | 1.06e-003 | 0.37 | -0.22 | 0.12 |
| GCSF | -0.44 | 0.14 | 0.12 | -0.13 | 0.22 |
| ICAM1 | -0.46 | 0.11 | 0.16 | -0.37 | -0.49 |
| BDNF | -0.73 * | -0.30 | -0.63 * | -0.52 | -0.68 * |

**Supplemental Figure 4. Processing delay exerts relatively greater changes in circulating cytokines as a function of group median.**

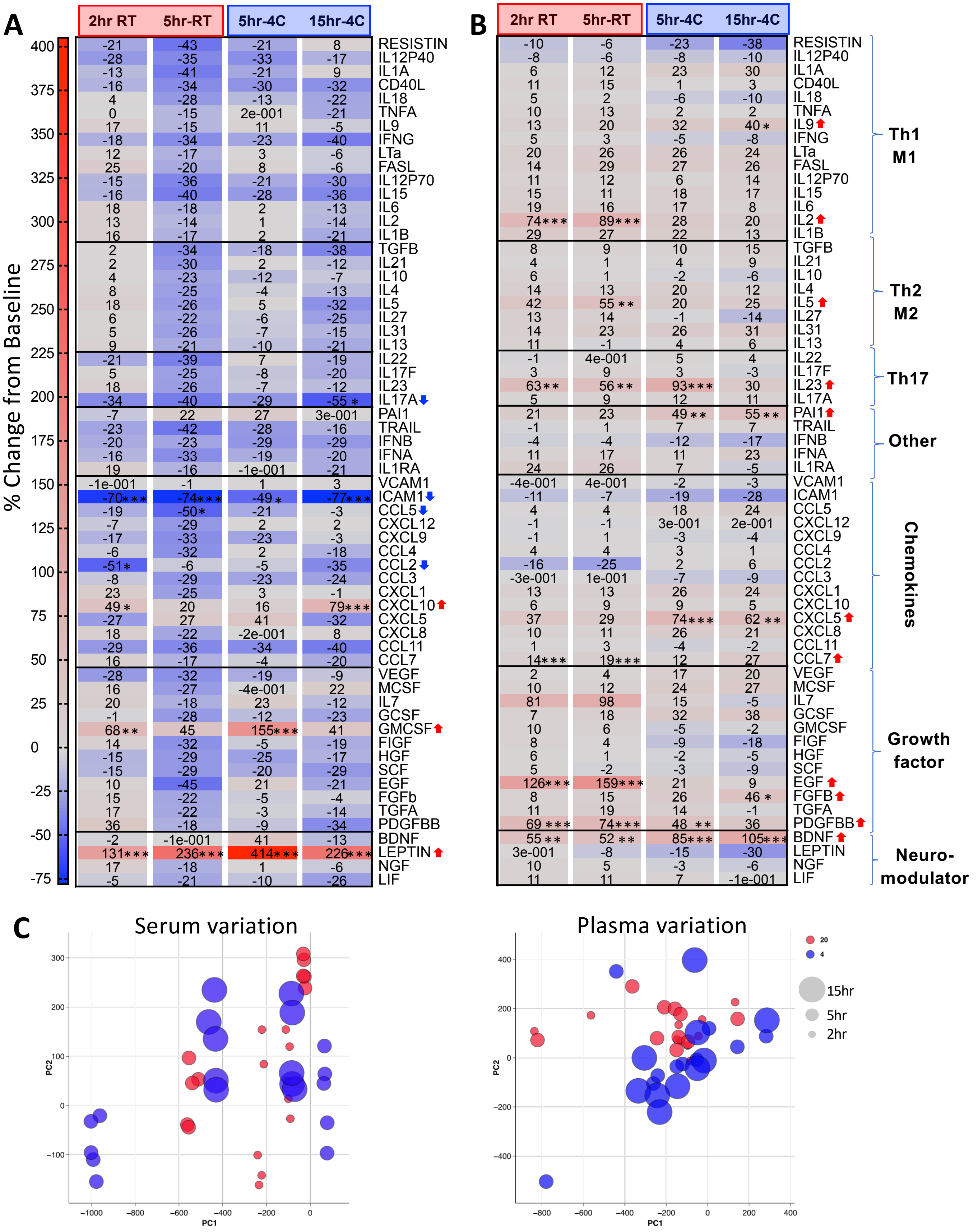

# Supplemental Figure 5. CyTOF gating schemes.

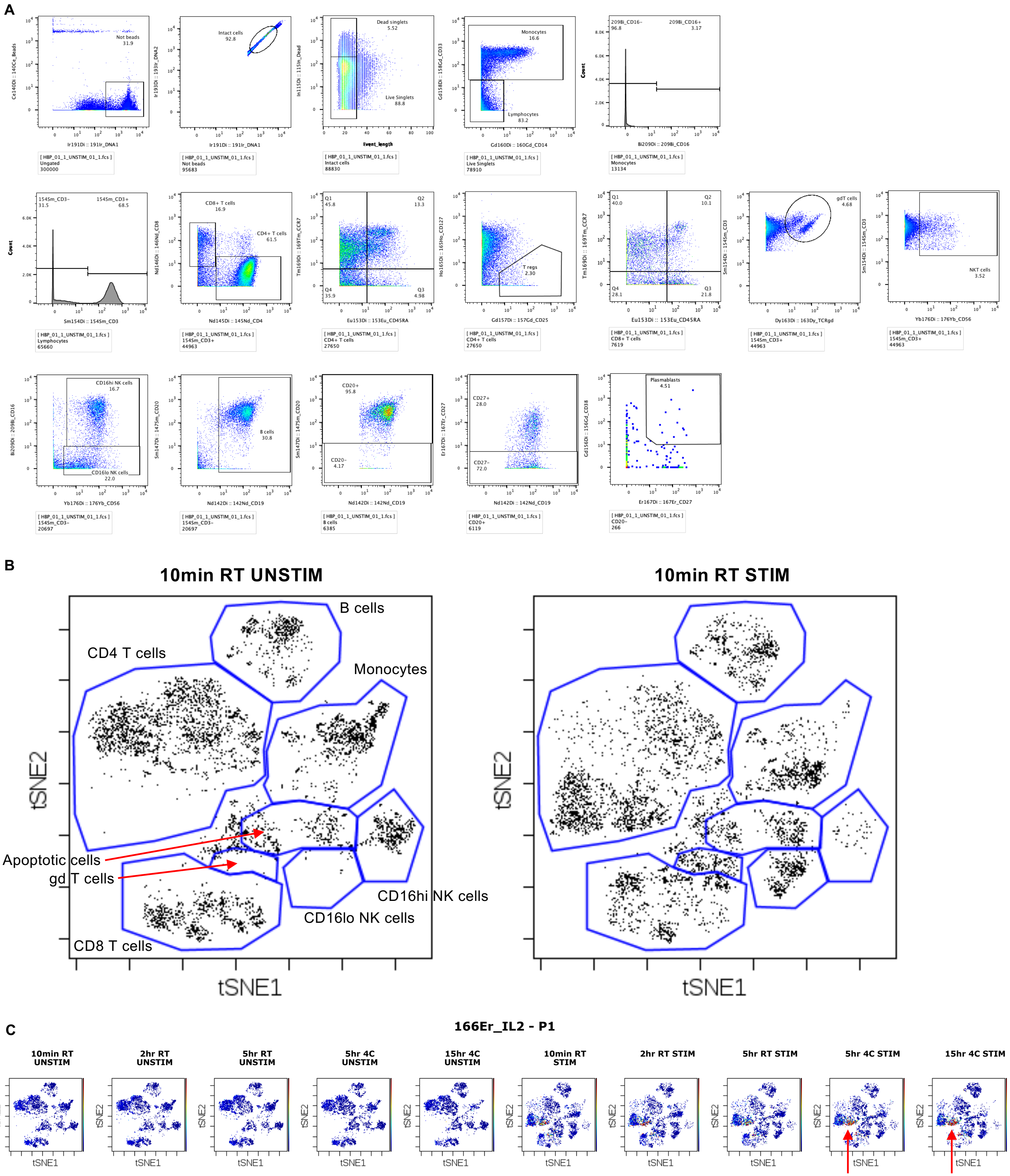

## Supplemental Figure 6. CyTOF viSNE analysis identifies significant differences between baseline and 15 h 4°C conditions.

A

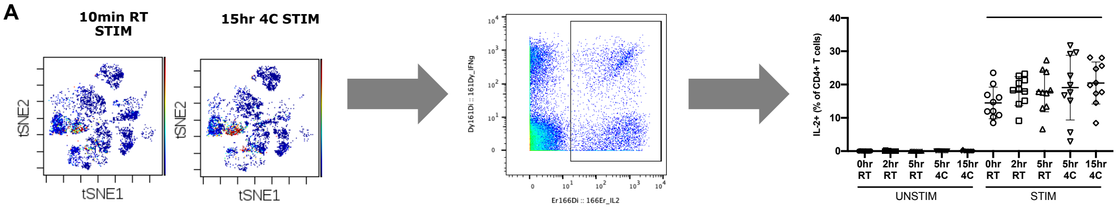


B

| Condition 1 | Condition 2 | Population | P-value | Mean % change (condition 1 to 2) |
|---|---|---|---|---|
| 0hr RT UNSTIM | 15hr 4C UNSTIM | Median HLA-DR of B cells | 0.0036 | 13.32 |
| | | HLA-DR+ (% of B cells) | 0.0156 | -2.08 |
| | | Median CTLA-4 of monocytes | 0.0374 | -23.15 |
| | | Median CD14 of monocytes | 0.0029 | -75.04 |
| | | CD14+ (% of monocytes) | 0.0001 | -34.37 |
| | | Median CD33 of monocytes | 0.0010 | -41.43 |
| | | CD33+ (% of monocytes) | 0.0015 | -28.73 |
| | | Median TNFa of CD4+ T cells | 0.0015 | -35.17 |
| | | Median CD36 of monocytes | 0.0007 | -55.25 |
| | | CD36+ (% of monocytes) | 0.0051 | -16.30 |
| | | Median IL2 of CD4+ T cells | 0.0180 | -7.55 |
| 0hr RT STIM | 15hr 4C STIM | Median HLA-DR of B cells | 0.0028 | 9.01 |
| | | HLA-DR+ (% of B cells) | 0.0290 | -1.88 |
| | | Median CTLA-4 of monocytes | 0.0009 | -48.18 |
| | | Median CD107a of monocytes | 0.0008 | -58.75 |
| | | CD107a+ (% of monocytes) | 0.0004 | -27.50 |
| | | Median CD14 of monocytes | 0.0007 | -85.96 |
| | | CD14+ (% of monocytes) | 0.0000 | -51.52 |
| | | Median IL10 of B cells | 0.0233 | -35.06 |
| | | IL10+ (% of B cells) | 0.0222 | -25.67 |
| | | Median CD33 of monocytes | 0.0047 | -32.28 |
| | | CD33+ (% of monocytes) | 0.0474 | 165.32* |
| | | Median MIP1b of CD16lo NK cells | 0.0027 | 129.21 |
| | | MIP1b+ (% of CD16lo NK cells) | 0.0417 | 15.08 |
| | | TNFa+ (% of CD4+ T cells) | 0.0290 | 25.88 |
| | | Median CD36 of monocytes | 0.0035 | -54.38 |
| | | CD36+ (% of monocytes) | 0.0006 | -24.71 |
| | | IL2+ (% of CD4+ T cells) | 0.0068 | 47.65 |

## Supplemental Figure 7. CyTOF CITRUS analysis identifies significant differences between baseline and 15 h 4°C conditions.

A

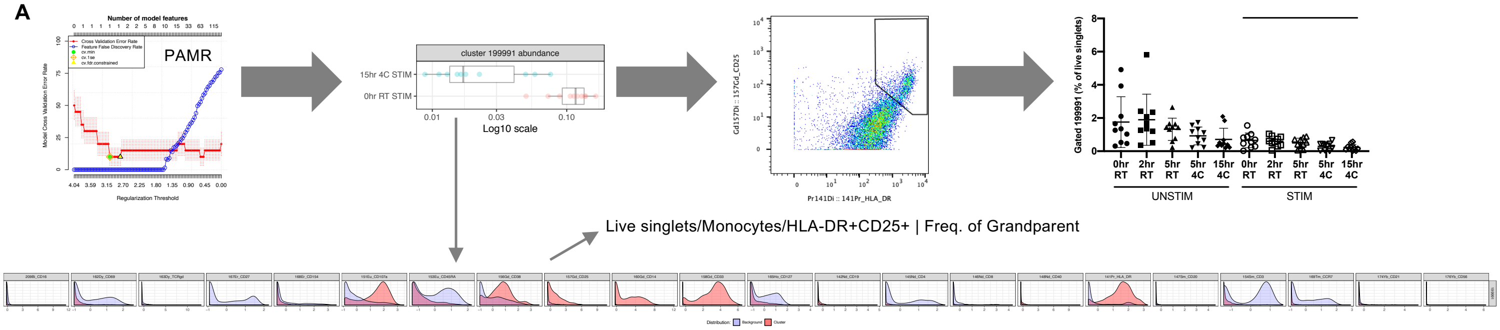


B

| Condition 1 | Condition 2 | Population | P-value | Mean % change (condition 1 to 2) |
|---|---|---|---|---|
| 0hr RT UNSTIM | 15hr 4C UNSTIM | CD45RA+CCR7+CD27+CD154+ CD8+ T cells (% of live singlets) | 0.0066 | -56.38 |
| | | CD45RA-CCR7+ (% of CD8+ T cells) | 0.0382 | -27.16 |
| | | CD45RA+CCR7+ (% of CD8+ T cells) | 0.0001 | -36.45 |
| | | CD45RA+CCR7- (% of CD8+ T cells) | 0.0012 | 84.49 |
| | | CD45RA-CCR7- (% of CD8+ T cells) | 0.0101 | 64.55 |
| | | HLA-DR+CD33+ (% of live singlets) | 0.0000 | -68.84 |
| | | Median PD-L1 of CD45RA-CCR7+CD107a+ CD4+ T cells | 0.0027 | -61.09 |
| | | Median PD-L1 of CD45RA+CCR7+CD107a+ CD4+ T cells | 0.0094 | -47.73 |
| | | Median PD-L1 of CD45RA-CCR7-CD107a+ CD4+ T cells | 0.0038 | -84.40 |
| | | Median granzyme B of CD33+HLA-DR+CD25+CD14+CD38+CD107a+CD16- cells | 0.0005 | -56.78 |
| | | Median CTLA-4 of HLA-DR+CD107a+ cells | 0.0004 | -52.27 |
| | | Median IL-4 of CD16hi NK cells | 0.0226 | -79.36 |
| | | Median CTLA-4 of CD16hi NK cells | 0.0022 | -40.79 |
| | | Median CTLA-4 of CD45RA+CCR7- CD8+ % cells | 0.0011 | -41.06 |
| | | Median CTLA-4 of CD33+HLA-DR+ cells | 0.0487 | -20.52 |
| | | Median CTLA-4 of B cells | 0.0190 | -24.85 |
| 0hr RT STIM | 15hr 4C STIM | HLA-DR+CD25+ (% of monocytes) | 0.0169 | -54.46 |

**Supplemental Figure 8. Independent analysis of CyTOF data with Astrolabe is consistent with other guided analysis approaches.**

A

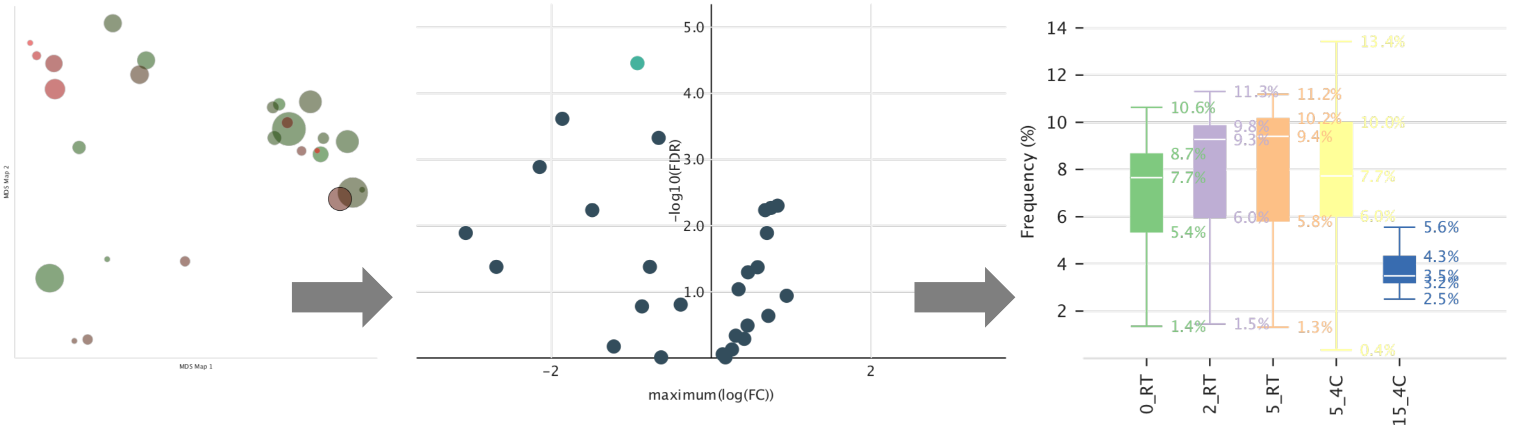


B

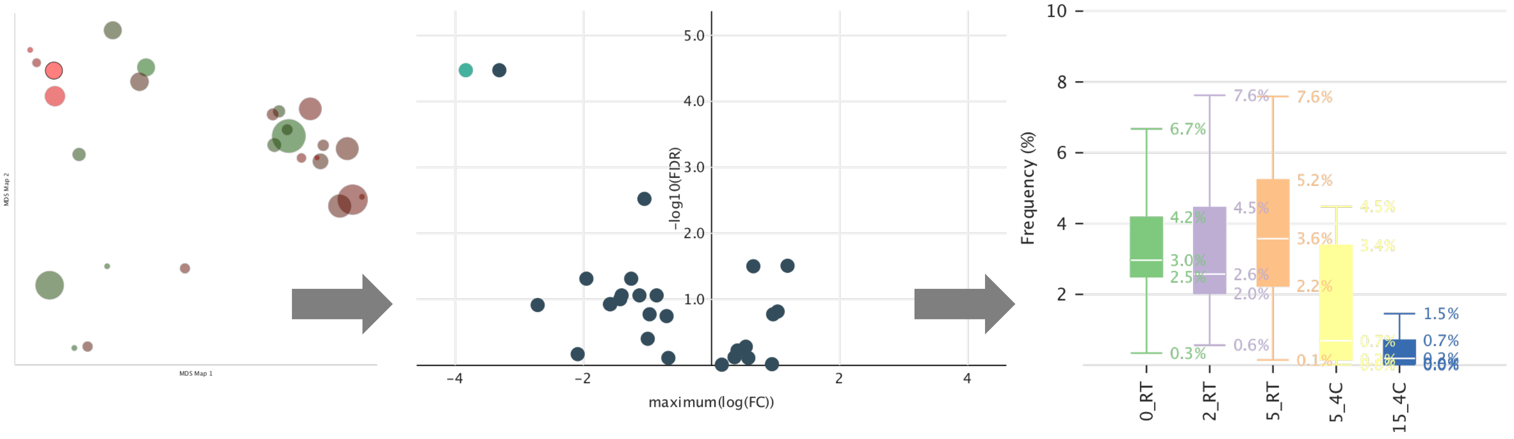

Supplement: Supplementary file 2 — Supplementary Figures. [file 41598_2020_74274_MOESM2_ESM.pdf]
